# Supplementary material for: Mussel-Inspired Lego Approach for Controlling the Wettability of Surfaces with Colorless Coatings
Source: Biomimetics (Basel). 2022 Dec 21;8(1):3. doi: 10.3390/biomimetics8010003 (PMC9844497; doi:10.3390/biomimetics8010003)
Supplement: Supplementary file 1 [file biomimetics-08-00003-s001.zip › biomimetics-2059709-supplementary.pdf]

Table of contents

S1. Synthesis of monomers **4a** and **4b** (Experimental part)	S3
S2. Synthesis of compounds **6a** and **6b** (Experimental part)	S6
S3. Polymerization of **4b** and **6b**	S9
S4. ^{1}H and ^{13}C NMR spectra of new compounds	S10
^{1}H and ^{13}C NMR spectra of compound **2**	S10
^{1}H and ^{13}C NMR spectra of compound **4a**	S11
^{1}H and ^{13}C NMR spectra of compound **4b**	S12
^{1}H and ^{13}C NMR spectra of compound **5**	S13
^{1}H and ^{13}C NMR spectra of compound **6a**	S14
^{1}H and ^{13}C NMR spectra of compound **6b**	S15
^{1}H and ^{13}C NMR spectra of compound **7**	S16
S5. IR spectra of polymeric materials	S17
IR spectrum of polymer **P4a**	S17
IR spectrum of polymer **P6a**	S17
IR spectrum of polymer **P4b**	S18
IR spectrum of polymer **P6b**	S18
S6. GPC spectra	S19
GPC spectra of building block **4b**	S19
GPC spectra of building block **6b**	S20
S7. Coating procedure	S21
S8. EDX spectra	S22
EDX of pristine and coated surfaces with **P4b** *via ex situ* polymerisation in solution	S22-S23
EDX of coated surfaces with **P4b** *via in situ* polymerisation	S24
S9. Characterization of coated textiles	S25

S1. Synthesis of monòmers 4a and 4b

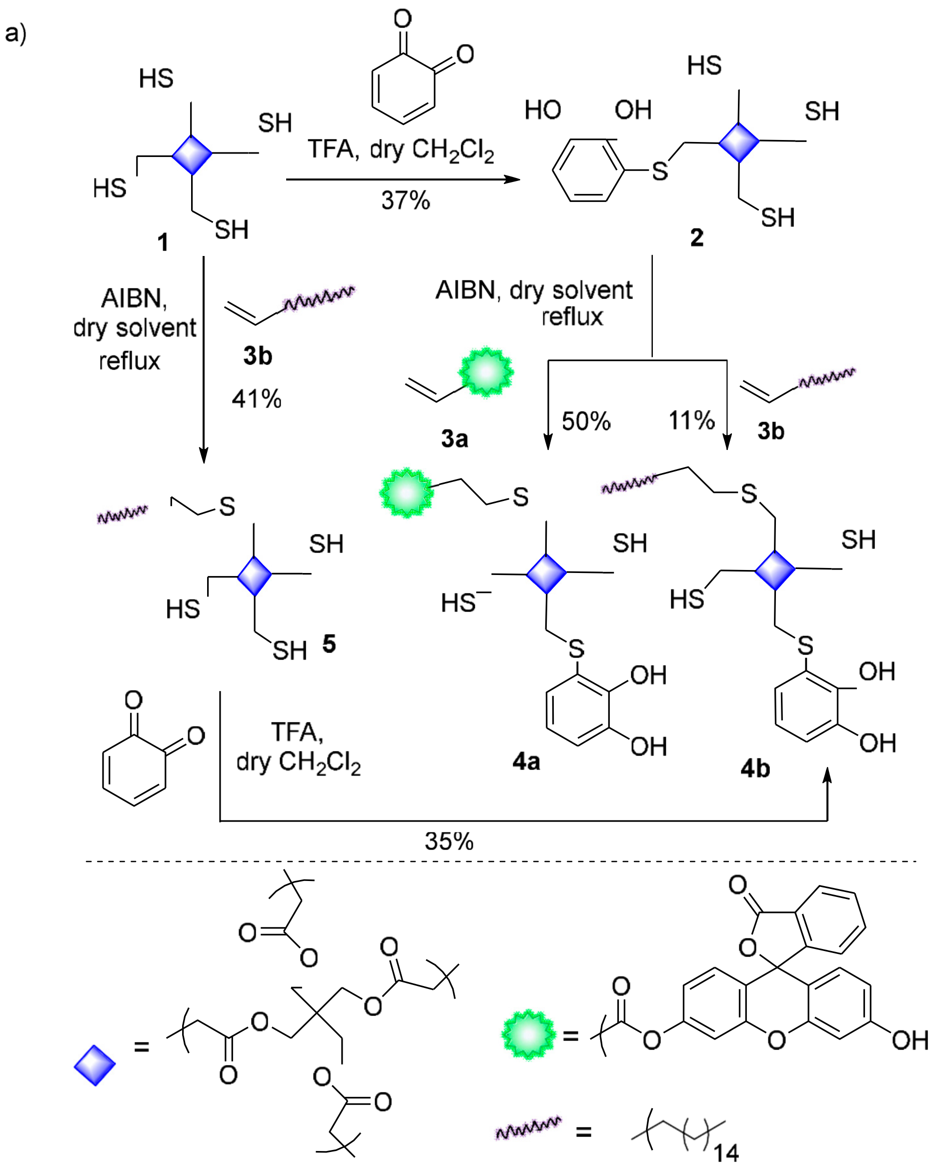

Scheme 1

3-[(3-sulfanylpropanoyl)oxy]-2,2-bis{[(3-sulfanylpropanoyl)oxy]methyl}propyl 3-[(2,3-dihydroxyphenyl)sulfanyl]propanoate, 2. A solution of pyrocatechol (571 mg, 5.21 mmol) in the minimum volume of Et_2O was added to a solution of $NaIO_4$ (1.393 g, 6.51 mmol) in H_2O (170 mL) cooled down to 0 °C. The final mixture was stirred for 10 min. The resulting *o*-benzoquinone was extracted with CH_2Cl_2 (3 x 170 mL) in quantitative yield. The organic extracts were dried with Na_2SO_4 anhydrous and filtered. In the meantime, a solution of pentaerythritol tetrakis(3-mercaptopropionate), **1**, (2.715 g, 5.53 mmol) in CH_2Cl_2 (8 mL) with trifluoroacetic acid (TFA) (1 mL, 13.32 mmol) was prepared under N_2 atmosphere. Finally, the solution containing *o*-benzoquinone was added into the solution containing **1**. The resulting mixture was stirred at rt under Ar atmosphere for 8 h in the dark. After this time, the solvent and TFA were removed under vacuum. The resulting oil was purified by column chromatography using hexane:EtOAc 7:3 as eluent affording *S*-catechol tris-thiol **2** as an oil (1.221 g,

2.05 mmol, 37% yield): ^{1}H NMR (400 MHz, $CDCl_3$) δ 6.92 (dd, *J* = 7.7, 1.6 Hz, 1H, H-6'), 6.87 (dd, *J* = 7.7, 1.6 Hz, 1H, H-4'), 6.73 (t, *J* = 7.7 Hz, 1H, H-5'), 4.16 (s, 8H, H-3'',H-1'',2xH-1'''), 2.93 (t, *J* = 7.1 Hz, 2H, H-3), 2.72 (m, 6H, H-H-3'',2xH-3^{V}), 2.64 (m, 6H, H-2''',2xH-2^{V}), 2.51 (t, *J* = 7.1 Hz, 2H, H-2), 1.60 (t, *J* = 8.2 Hz, 3H, -SH). ^{13}C NMR (100 MHz, $CDCl_3$) δ 171.6/171.5 (4xC=O), 144.7/144.2 (C-2'/C-3'), 126.4 (C-5'), 120.8 (C-6'), 117.7 (C-4'), 116.8 (C-1'), 62.0 (C-3'',C-1'',2xC-1''''), 41.9 (C-2''), 38.1 (C-2''',2xC-2^{V}), 33.6 (C-2), 30.8 (C-3), 19.5 (C-3''',2xC-3^{V}). HRMS (HR-EI) calcd. for $[C_{23}H_{32}O_{10}S_4Na]^+$: 619.0777; found: 619.0776.

3-({3-[(3-{[6-hydroxy-9*H*-spiro(3-oxo-isobenzofuran-1-yl)xanthen-3-yl]oxy}-3-oxopropyl) sulfanyl]propanoyl}oxy)-2,2-bis{[(3-sulfanylpropanoyl)oxy]methyl}propyl 3-[(2,3-dihydroxyphenyl)sulfanyl]propanoate, 4a. To a solution of fluorescein *O*-acrylate (150 mg, 0.39 mmol) in dry CH_3CN (12 mL), AIBN (36 mg, 0.22 mmol) was added under N_2 atmosphere. The mixture was heated at reflux temperature and a solution of *S*-catechol tris-thiol **2** (450 mg, 0.75 mmol) dry CH_3CN (5 mL) was added dropwise for 1 h. The reaction mixture was heated at reflux temperature overnight. The cooled reaction was concentrated under reduced pressure to afford an oil which was purified by column chromatography using a gradient of hexanes:EtOAc. The fraction of interest was crystallised with Et_2O to afford the final pure compound **4a** as an orange powder (192 mg, 0.19 mmol, 50% yield): ^{1}H NMR (400 MHz, $(CD_3)_2CO$) δ 8.01 (m, 1H, H-Ar$_{fluores}$), 7.79 (m, 1H, H-Ar$_{fluores}$), 7.72 (m, 1H, H-Ar$_{fluores}$), 7.32-7.17 (br. s, 3H, H-6', H-5', H-4'), 6.95-6.87 (br. s, 3H, H-Ar$_{fluores}$), 6.79 (br. s, 1H, H-Ar$_{fluores}$), 6.74 (s, 1H, H-Ar$_{fluores}$), 6.66 (s, 1H, H-Ar$_{fluores}$), 6.63 (br. s, 1H, H-Ar$_{fluores}$), 4.26 (br. s, 8H, H-3'',H-1'',2xH-1^{V}), 3.17-2.61 (br.m, 20 H, H-2, H-3, H-2'', H-3'',2xH-2'''',2xH-3'''',H-2^{VI},H-3^{VI}), 1.61 (m, 2H, SH). ^{13}C NMR (100.6 MHz, $(CD_3)_2CO$) δ 170.8/171.5/171.3/171.1/170.8/168.8 (6xC=O), 159.9 (C-Ar$_{fluores}$), 159.5 (C-Ar$_{fluores}$), 153.1 (C-Ar$_{fluores}$), 152.9 (C-Ar$_{fluores}$), 152.4 (C-Ar$_{fluores}$), 152.3 (C-Ar$_{fluores}$), 152.1 (C-Ar$_{fluores}$), 151.7 (C-Ar$_{fluores}$), 135.4/135.1 (C-2'/C-3'/C-Ar$_{fluores}$), 130.1 (C-Ar$_{fluores}$), 129.2 (C-Ar$_{fluores}$), 129.1 (C-Ar$_{fluores}$), 126.6 (C-5'), 124.7/124.4/124.1 (C-1'/C-6'/C-Ar$_{fluores}$), 117.8/112.8/112.3/110.7/110.3 (C-4'/4xC-Ar$_{fluores}$), 102.5 (C-Ar$_{fluores}$), 69.1/62.3/62.2 (C-3''/C-1''/C-1^{V}), 43.4/33.7/27.6/25.7 (C-2/C-3/C-2'''/C-2''''), 25.7 (C-3''',C-3''''). HRMS (HR-EI) calcd. for $[C_{46}H_{47}O_{16}S_4]^+$: 983.1742; found: 983.1747.

3-{[3-(octadecylsulfanyl)propanoyl]oxy}-2,2-bis{[(3-sulfanylpropanoyl)oxy]methyl} propyl 3-[(2,3-dihydroxyphenyl)sulfanyl]propanoate, 4b.

Method A. AIBN (31 mg, 0.19 mmol) and S-catechol tris-thiol **2** (535 mg, 0.90 mmol) were dissolved in dry toluene(10 mL), and 1-octadecene (291 µL, 0.81 mmol) were added. The mixture was heated at reflux temperature under Ar atmosphere for 24 h. The cooled reaction was concentrated under reduced pressure to afford a yellow oil which was purified by column chromatography using a gradient of hexane:EtOAc as eluent. The final compound **4b** was obtained as a colourless oil (75 mg, 0.09 mmol, 11% yield).

Method B. Pentaerythritol tetrakis(3-mercaptopropionate) (2.541 g, 5.20 mmol) was dissolved in dry toluene (30mL). Then, a solution of AIBN (180 mg, 1.10 mmol) in the minimum volume of dry toluene and 1- octadecene (1.8 mL, 5.03 mmol) were added. The mixture was heated at 80 °C under Ar atmosphere for 8 h. The cooled reaction was concentrated under reduced pressure to afford a yellow oil. The mixture was purified by column chromatography using a gradient of hexane:EtOAc as eluent to obtain compound **5,** 3-[(3-sulfanylpropanoyl)oxy]-2,2-bis{[(3-sulfanylpropanoyl)oxy]methyl}propyl 3- (octadecylsulfanyl)propanoate, as a colourless oil (1.519 g, 2.04 mmol, 41% yield).

A solution of pyrocatechol (110 mg, 1.00 mmol) in the minimum volume of Et_2O was added to a solution of $NaIO_4$ (265 mg, 2.89 mmol) in H_2O (34 mL) cooled down to 0 °C. The final mixture was stirred for 10 min. The resulting *o*-benzoquinone was extracted with dry CH_2Cl_2 (3 x 34 mL) in quantitative yield. The organic extracts were dried with Na_2SO_4 anhydrous and filtered. In the meantime, a solution of aliphatic chain precursor *S*-C_{18}-functionalised tris-thiol 5 (693 mg, 0.93 mmol) in dry CH_2Cl_2 (2.5 mL) with TFA (0.22 mL, 2.93 mmol) was prepared under an N_2 atmosphere. Then, the solution containing the *o*-benzoquinone was added, and the resulting mixture was stirred at rt under N_2 atmosphere for 8 h in the dark. After this time, the solvent and TFA were removed under vacuum. The resulting oil was purified by column chromatography using a gradient of hexane:EtOAc as eluent to afford S,S'-catechol-C_{18}-bis-thiol **4b** as a colourless oil (268 mg, 0.32 mmol, 35% yield).

Compound **5**. ^{1}H NMR (400 MHz, $CDCl_3$) δ 4.16 (s, 8H, H-1',H-3',2xH-1''''), 2.73 (m, 6H, H-3'',2xH-3''''), 2.64 (m, 8H, H-2,H-2'',2xH-2''''), 2.48 (t, *J* = 7.1 Hz, 4H, H-3,H-1^{V}), 1.60 (t, *J* = 7.8 Hz, 3H, -SH), 1.53 (q, *J* = 7.2 Hz, 2H, H-2^{V}), 1.42-1.08 (br. s, 30H, H-3^{V}-H-17^{V}), 0.84 (t, *J* = 7.1 Hz, 3H, H-18^{V}). ^{13}C NMR (100 MHz, $CDCl_3$) δ 171.9/171.5/171.4/171.0 (C=O), 62.1 (C-1',C-3',2xC-1''''), 41.9 (C-2'), 38.3 (C-2,C-2'',2xC-2''''), 32.1 (C-1^{V},C-3), 29.7 (C-2^{V}-C-16^{V}), 26.9 (C-3^{V}), 22.7 (C-17^{V}), 19.7 (C-3'',2xC-3''''), 14.1 (C-18^{V}). HRMS (HR-EI) calcd. for $[C_{35}H_{64}O_8S_4Na]^+$: 763.3379; found: 763.3382.

Compound **4b**. ^{1}H NMR (400 MHz, $CDCl_3$) δ 7.00 (dd, *J* = 8.0, 1.6 Hz, 1H, H-6'), 6.95 (dd, *J* = 8.0, 1.6 Hz, 1H, H-4'), 6.81 (td, *J* = 8.0, 2.5 Hz, 1H, H-5'), 4.20 (s, 8H, H-1'',H-3'',2xH-1^{V}), 3.03 (t, *J* = 7.1 Hz, 2H, H-3), 2.73 (m, 6H, H-3''',2xH-3''''), 2.64 (m, 6H, H-2''',2xH-2''''), 2.56 (t, *J* = 7.1 Hz, 4H, H-2, H-1^{VI}), 1.66 (t, *J* = 8.3 Hz, 2H, -SH), 1.60 (q, *J* = 7.2 Hz, 2H, H-2^{VI}), 1.43-1.18 (br. s, 30H, H-3^{VI}-H-17^{VI}), 0.90 (t, *J* = 7.2 Hz, 3H, H-18^{VI}). ^{13}C NMR (100 MHz, $CDCl_3$) δ 172.0/171.6/ 171.1/170.8 (C=O), 145.1/144.2 (C-2'/C-3'), 127.0 (C-5'), 126.0 (C-6'), 121.2 (C-1'), 117.2 (C-4'), 62.6/62.4 (C-1''/C-3''/C-1^{V}), 42.5 (C-2''), 38.3 (C-2''',2xC-2''''), 31.9 (C-2,C-3,C-1^{VI}), 29.7 (C-2^{VI}-C-16^{VI}), 26.9 (C-3'',2xC-3'''), 22.7 (C-17^{VI}), 14.1 (C-18^{VI}). HRMS (HR-EI) calcd. for $[C_{41}H_{68}O_{10}S_4Na]^+$: 872.3604; found: 872.3593.

3-({3-[(3-{[6-hydroxy-9*H*-spiro(3-oxo-isobenzofuran-1-yl)xanthen-3-yl]oxy}-3-oxopropyl)sulfanyl]propanoyl}oxy)-2,2-bis{[(3-sulfanylpropanoyl)oxy]methyl}propyl 3-[(2-phenylethenyl)sulfanyl]propanoate, 6a (39% Yield). ^{1}H NMR (360 MHz, $(CD_3)_2CO$) δ 8.01 (m, 2H, H-$Ar_{fluores}$), 7.86-7.67 (br. m, 3H, H-$Ar_{fluores}$), 7.38-7.12 (br. m, 5H, H-2''', H-3''', H-4''',H-5''',H-6'''), 6.96-6.82 (br. s, 2H, H-$Ar_{fluores}$), 6.80 (br. s, 1H, H-$Ar_{fluores}$), 6.74-6.62 (br. s, 2H, H-$Ar_{fluores}$), 4.25 (br. s, 8H, H-1',H-3',2xH-1^{VI}), 3.17-2.65 (br. m, 24H, H-2,H-3,H-1'',H-2'',H-2'''',H-3'''',2xH-2^{V},2xH-3^{V},H-2^{VII}, H-3^{VII}), 1.67 (t, *J* = 8.0 Hz, 2H, -SH). ^{13}C NMR (90.5 MHz, $(CD_3)_2CO$) δ 172.0/171.5/171.3/171.1/170.8 (C=O), 168.8 (C-$Ar_{fluores}$), 159.6 (C-$Ar_{fluores}$), 153.1 (C-$Ar_{fluores}$), 152.9 (C-$Ar_{fluores}$), 152.5 (C-$Ar_{fluores}$), 152.3 (C-$Ar_{fluores}$), 152.2 (C-$Ar_{fluores}$), 141.4 (C-1'''), 135.2 (C-3''',C-5'''), 130.7 (C-$Ar_{fluores}$), 130.1 (C-$Ar_{fluores}$), 129.8 (C-2''',C-6'''), 129.3 (C-$Ar_{fluores}$), 129.1 (C-$Ar_{fluores}$), 128.8 (C-4'''), 124.7 (C-$Ar_{fluores}$), 112.8 (C-$Ar_{fluores}$), 112.4 (C-$Ar_{fluores}$), 110.8 (C-$Ar_{fluores}$), 110.3 (C-$Ar_{fluores}$), 102.5 (C-$Ar_{fluores}$), 102.4 (C-$Ar_{fluores}$), 82.4 (C-$C_{fluores}$), 62.9 (C-1',C-3',2xC-1^{VI}), 42.6 (C-2'), 38.5/36.5/35.1/34.9/33.9/33.6 (C-2/2xC-2^{V}/C-2''''/C-2^{VII}/C-1''/C-2''), 26.8/24.8/22.7/19.7 (C-3''''/2xC-3^{V}/C-3^{VI}/C-3). HRMS (HR-EI) calcd. for $[C_{48}H_{51}O_{14}S_4]^+$: 979.2141; found: 979.2161.

3-{[3-(octadecylsulfanyl)propanoyl]oxy}-2,2-bis{[(3-sulfanylpropanoyl)oxy]methyl} propyl 3-[(2-phenylethenyl)sulfanyl]propanoate, 6b (75% yield). ^{1}H NMR (400 MHz, CD_2Cl_2) δ 7.31-7.16 (m, 5H, H-2''', H-6''',H-3''', H-5''',H-4'''), 4.15 (br. s, 8H, H-1',H-3'',2xH-1^{VI}), 2.87 (m, 6H, H-3,H-1'',H-2''), 2.75 (m, 8H, 2xH-3^{V},H-3'''',H-1^{VII}), 2.62 (m, 6H, H-2'''',2xH-2^{V}), 2.50 (t, *J* = 7.2 Hz, 2H, H-2), 1.65 (t, *J* = 8.3 Hz, 2H, -SH), 1.56 (q, *J* = 7.0 Hz, 2H, H-2^{VII}), 1.30-1.17 (br. s, 30H, H-3^{VII}-H-17^{VII}), 0.87 (t, *J* = 7.0 Hz, 3H, H-18^{VII}). ^{13}C NMR (100 MHz, $CDCl_3$) δ 171.9/171.4/171.3/171.1 (C=O), 140.4 (C-1'''), 128.6/128.4 (C-2'''/C-6'''/C-3'''/C-5'''), 126.6 (C-4'), 62.6 (C-1',C-3',2xC-1^{VI}), 42.0 (C-2'), 38.5 (C-2), 36.2 (C-2''',2xC-2^{V}), 34.6/33.9 (C-1''/C-2''), 32.0 (C-1^{VII}), 29.9/29.5 (C-2^{VII}-C-16^{VII}), 27.0 (C-3), 22.9 (C-3''',2xC-3^{V}), 20.0 (C-17^{VII}), 14.5 (C-18^{VII}). HRMS (HR-EI) calcd. for $[C_{43}H_{72}O_8S_4]^+$: 844.2897; found: 844.4110.

S2. Synthesis of compounds 6a and 6b

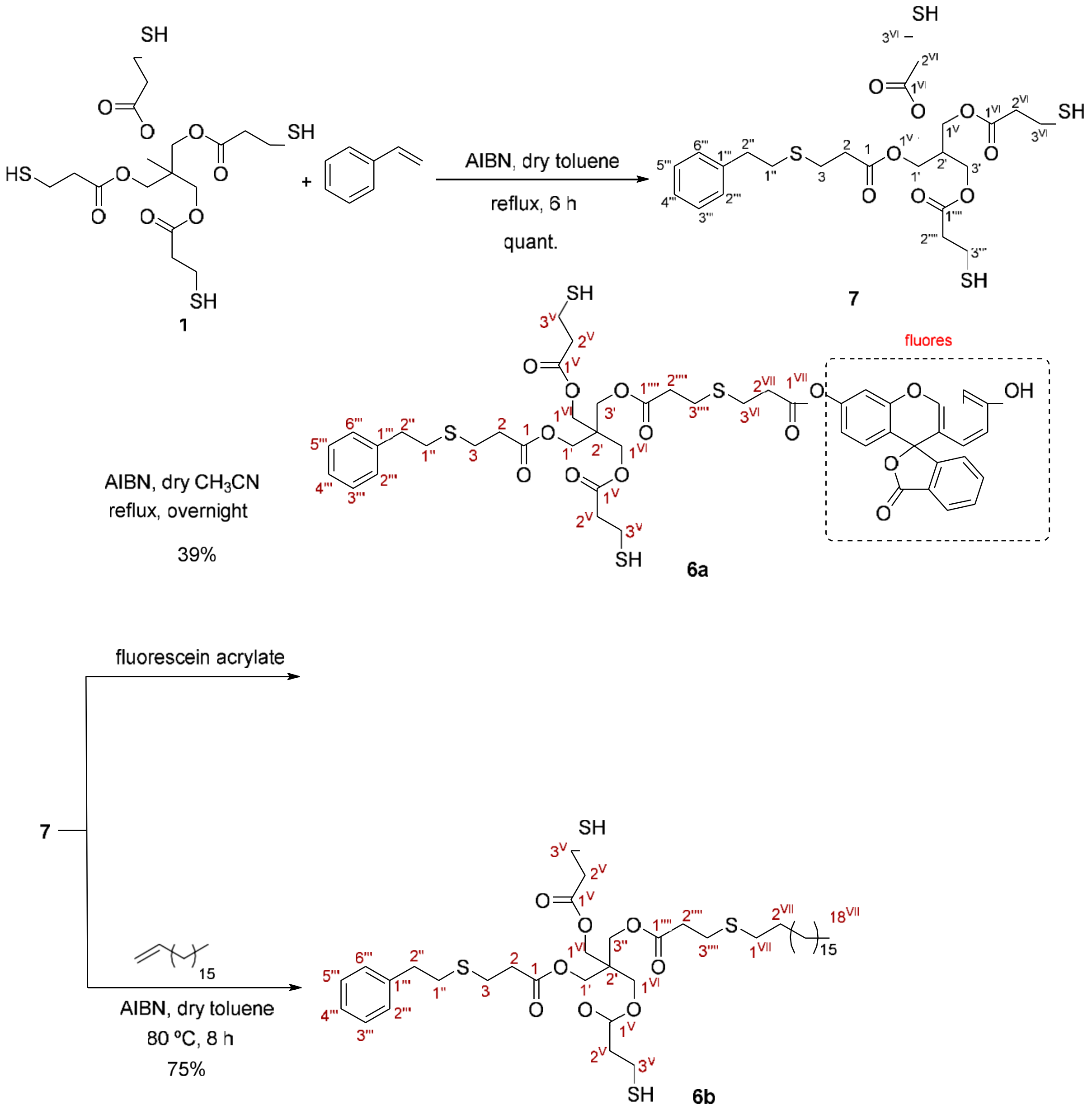

Scheme 2

3-[(3-sulfanylpropanoyl)oxy]-2,2-bis{[(3-sulfanylpropanoyl)oxy]methyl}propyl 3-[(2-phenylethenyl)sulfanyl]propanoate, 7. A solution of pentaerythritol tetrakis(3-mercaptopropionate) (436 mg, 0.89 mmol) in dry toluene (5 mL) was added into a Schlenk containing AIBN (31 mg, 0.19 mmol). Afterwards, styrene (100 µL, 0.87 mmol) was added. The mixture was heated at reflux temperature under Ar atmosphere. After 6 h, the cooled reaction was concentrated under reduced pressure to afford **7** as a colourless oil (513 mg, 0.87 mmol, quant.): ^{1}H NMR (360 MHz, CD_2Cl_2) δ 7.30-7.16 (m, 5H, H-2′′′,H-3′′′,H-4′′′,H-5′′′,H-6′′′), 4.16 (br. s, 8H, H-3′,H-1′,2xH-1^{V}), 2.86 (t, *J* = 7.2 Hz, 2H, H-3), 2.72 (m,10H, H-1′′, H-2′′, H-3′′′′,2xH-3^{VI}), 2.64 (m, 8H, H-2′′′′, H-2,2xH-2^{VI}), 1.66 (t, *J* = 8.2 Hz, 3H, -SH). ^{13}C NMR (90.5 MHz, CD_2Cl_2) δ 171.8/171.5/171.3/171.0 (C=O), 140.8 (C-1′′), 128.5/128.4 (C-2′′′/C-6′′′/C-3′′′/C-5′′′), 126.3 (C-4′′′), 62.6 (C-3′,C-1′,2xC-1^{V}), 41.9 (C-2′), 38.3 (C-2′′′′,C-2^{VI}), 36.0 (C-2), 34.8/33.6 (C-1′/C-2′), 26.9 (C-3), 19.6 (C-3′′′′,C-3^{VI}). HRMS (HR-EI) calcd. for $[C_{25}H_{38}O_9S_4]^+$: 610.1335; found: 610.1399.

3-({3-[(3-{[6-hydroxy-9*H*-spiro(3-oxo-isobenzofuran-1-yl)xanthen-3-yl]oxy}-3-oxopropyl)sulfanyl]propanoyl}oxy)-2,2-bis{[(3-sulfanylpropanoyl)oxy]methyl}propyl 3-[(2-phenylethenyl)sulfanyl]propanoate, 6a. A solution of *S*-styrene tris-thiol **7** (152 mg, 0.26 mmol) in dry CH_3CN (1 mL) was added into a Schlenk containing AIBN (10 mg, 0.06 mmol). Then, a solution of fluorescein *O*-acrylate (100 mg, 0.26 mmol) in dry CH_3CN (2 mL) was added. The mixture was heated at reflux temperature under Ar atmosphere overnight. The cooled reaction was concentrated under reduced pressure to afford a yellow oil which was purified by column chromatography using a gradient of hexane:EtOAc as eluent to afford compound **6a** as an orange solid (99 mg, 0.10 mmol, 39% yield): ^{1}H NMR (360 MHz, $(CD_3)_2CO$) δ 8.01 (m, 2H, H-Ar$_{fluores}$), 7.86-7.67 (br. m, 3H, H-Ar$_{fluores}$), 7.38-7.12 (br m, 5H, H-2′′′, H-3′′′, H-4′′′,H-5′′′,H-6′′′), 6.96-6.82 (br. s, 2H, H-Ar$_{fluores}$), 6.80 (sbr. , 1H, H-Ar$_{fluores}$), 6.74-6.62 (br. s, 2H, H-Ar$_{fluores}$), 4.25 (br. s, 8H, H-1′,H-3′,2xH-1^{VI}), 3.17-2.65 (br. m, 24H, H-2,H-3,H-1′′,H-2′′,H-2′′′′,H-3′′′′,2xH-2^{V},2xH-3^{V},H-2^{VII}, H-3^{VII}), 1.67 (t, *J* = 8.0 Hz, 2H, -SH). ^{13}C NMR (90.5 MHz, $(CD_3)_2CO$) δ 172.0/171.5/171.3/171.1/170.8 (C=O), 168.8 (C-Ar$_{fluores}$), 159.6 (C-Ar$_{fluores}$), 153.1 (C-Ar$_{fluores}$), 152.9 (C-Ar$_{fluores}$), 152.5 (C-Ar$_{fluores}$), 152.3 (C-Ar$_{fluores}$), 152.2 (C-Ar$_{fluores}$), 141.4 (C-1′′′), 135.2 (C-3′′′,C-5′′′), 130.7 (C-Ar$_{fluores}$), 130.1 (C-Ar$_{fluores}$), 129.8 (C-2′′′,C-6′′′), 129.3 (C-Ar$_{fluores}$), 129.1 (C-Ar$_{fluores}$), 128.8 (C-4′′′), 124.7 (C-Ar$_{fluores}$), 112.8 (C-Ar$_{fluores}$), 112.4 (C-Ar$_{fluores}$), 110.8 (C-Ar$_{fluores}$), 110.3 (C-Ar$_{fluores}$), 102.5 (C-Ar$_{fluores}$), 102.4 (C-Ar$_{fluores}$), 82.4 (C-C$_{fluores}$), 62.9 (C-1′,C-3′,2xC-1^{VI}), 42.6 (C-2′), 38.5/36.5/35.1/34.9/33.9/33.6 (C-2/2xC-2^{V}/C-2′′′′/C-2^{VII}/C-1′′/C-2′′), 26.8/24.8/22.7/19.7 (C-3′′′′/2xC-3^{V}/C-3^{VI}/C-3).HRMS (HR-EI) calcd. for $[C_{48}H_{51}O_{14}S_4]^+$: 979.2141; found: 979.2161.

3-{[3-(octadecylsulfanyl)propanoyl]oxy}-2,2-bis{[(3-sulfanylpropanoyl)oxy]methyl} propyl 3-[(2-phenylethenyl)sulfanyl]propanoate, 6b. A solution of AIBN (48 mg, 0.29 mmol) in dry toluene (8 mL) and 1-octadecene (0.4 mL, 1.12 mmol) were added to a solution of *S*-styrene tris-thiol **7** (740 mg, 1.25 mmol) in dry toluene (5 mL). The reaction mixture was heated at 80 °C under Ar

atmosphere for 8 h. The cooled reaction was concentrated under reduced pressure to afford a yellowish oil which was purified by column chromatography using a gradient of hexanes:EtOAc as eluent to obtain compound **6b** as a colourless oil (696 mg, 0.82 mmol, 75% yield): ^{1}H NMR (400 MHz, CD_2Cl_2) δ 7.31-7.16 (m, 5H, H-2''', H-6''',H-3''', H-5''',H-4'''), 4.15 (br. s, 8H, H-1',H-3'',2xH-1^{VI}), 2.87 (m, 6H, H-3,H-1'',H-2''), 2.75 (m, 8H, 2xH-3^{V},H-3'''',H-1^{VII}), 2.62 (m, 6H, H-2'''',2xH-2^{V}), 2.50 (t, *J* = 7.2 Hz, 2H, H-2), 1.65 (t, *J* = 8.3 Hz, 2H, -SH), 1.56 (q, *J* = 7.0 Hz, 2H, H-2^{VII}), 1.30-1.17 (br. s, 30H, H-3^{VII}-H-17^{VII}), 0.87 (t, *J* = 7.0 Hz, 3H, H-18^{VII}). ^{13}C NMR (100 MHz, $CDCl_3$) δ 171.9/171.4/171.3/171.1 (C=O), 140.4 (C-1‘‘‘), 128.6/128.4 (C-2‘‘‘/C-6‘‘‘/C-3‘‘‘/C-5‘‘‘), 126.6 (C-4‘), 62.6 (C-1‘,C-3‘,2xC-1^{VI}), 42.0 (C-2‘), 38.5 (C-2), 36.2 (C-2‘‘‘,2xC-2^{V}), 34.6/33.9 (C-1‘‘/C-2‘‘), 32.0 (C-1^{VII}), 29.9/29.5 (C-2^{VII}-C-16^{VII}), 27.0 (C-3), 22.9 (C-3‘‘‘,2xC-3^{V}), 20.0 (C-17^{VII}), 14.5 (C-18^{VII}). HRMS (HR-EI) calcd. for $[C_{43}H_{72}O_8S_4]^+$: 844.2897; found: 844.4110.

S3. Polymerization of 4b and 6b

Table S1. Polymerization's degree of **P4b** and **P6b** obtained by ^{1}H NMR, DOSY NMRexperiments and GPC.

Derivative	^{1}H NMR	DOSY	GPC			
			IR		UV	
			Mn	Mw	Mn	Mw
**P4b**	2-4 units	6/7 units	4017 Da<br>4/5 units	6013 Da<br>7 units	-	-
			IR		UV	
			Mn	Mw	Mn	Mw
**P6b**	dimer-trimer	< 10 units	3167 Da<br>tetramer	3651 Da<br>tetramer	3637 Da<br>tetramer	4144<br>5 units

S4. ^{1}H and ^{13}C NMR spectra

3-[(3-sulfanylpropanoyl)oxy]-2,2-bis{[(3-sulfanylpropanoyl)oxy]methyl}propyl 3-[(2,3-dihydroxyphenyl)sulfanyl]propanoate, 2

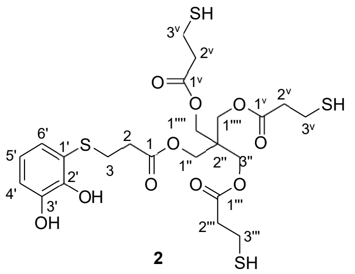

2

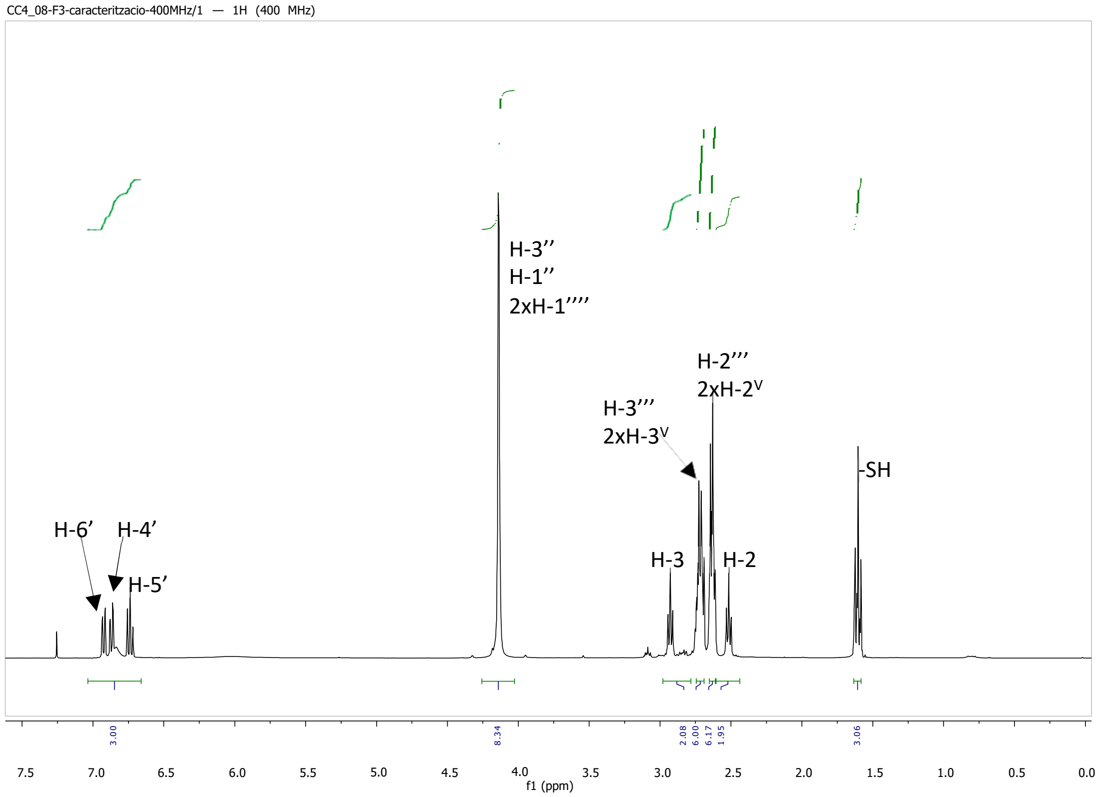

Figure S1. ^{1}H NMR (400MHz, $CDCl_3$) of compound **2**

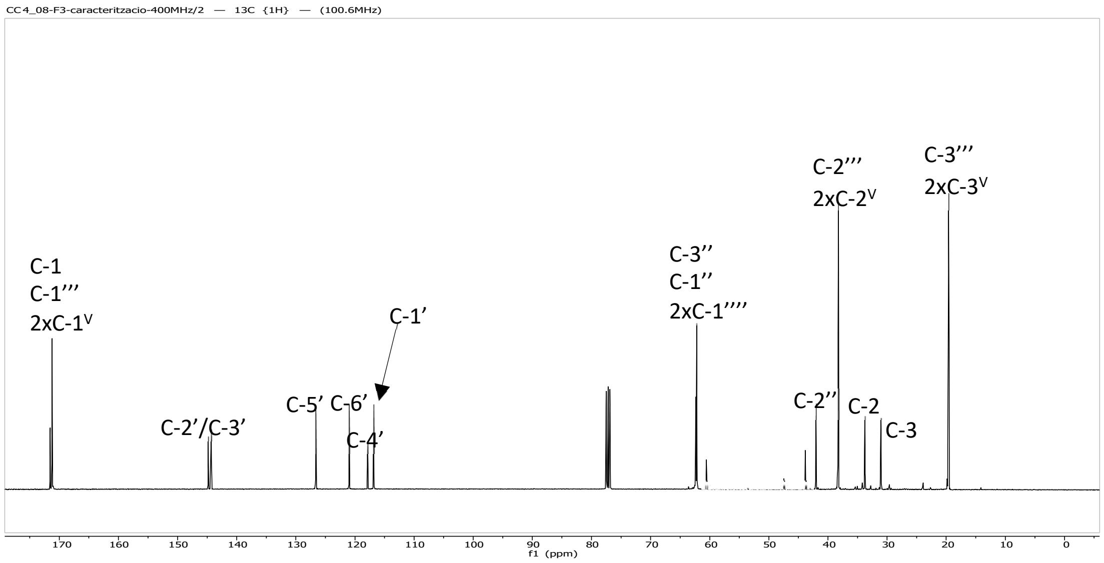

Figure S2. ^{13}C NMR (100MHz, $CDCl_3$) of compound **2**

3-({3-[(3-{[6-hydroxy-9*H*-spiro(3-oxo-isobenzofuran-1-yl)xanthen-3-yl]oxy}-3-oxopropyl)sulfanyl]propanoyl}oxy)-2,2-bis{[(3-sulfanylpropanoyl)oxy]methyl}propyl 3-[(2,3-dihydroxyphenyl)sulfanyl]propanoate, 4a

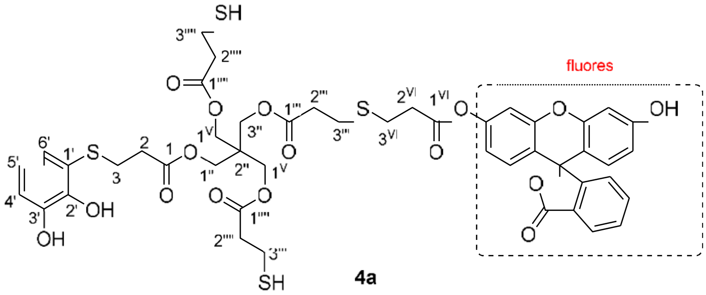

4a

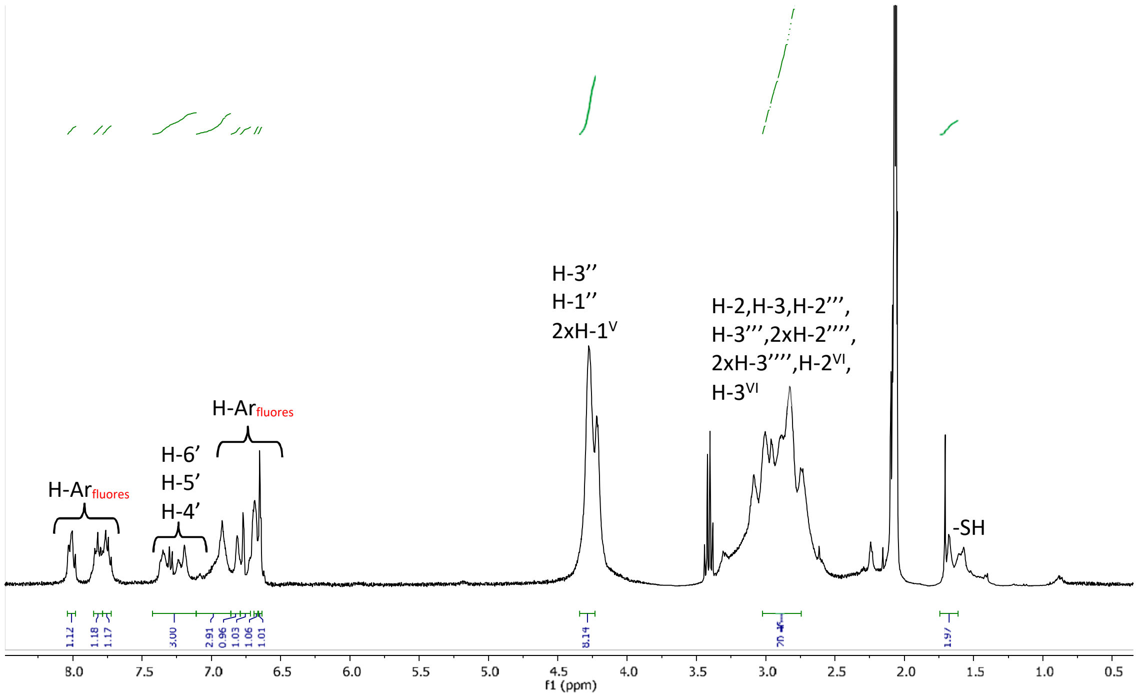

Figure S3. ^{1}H NMR (400MHz, $CDCl_3$) of compound **4a**

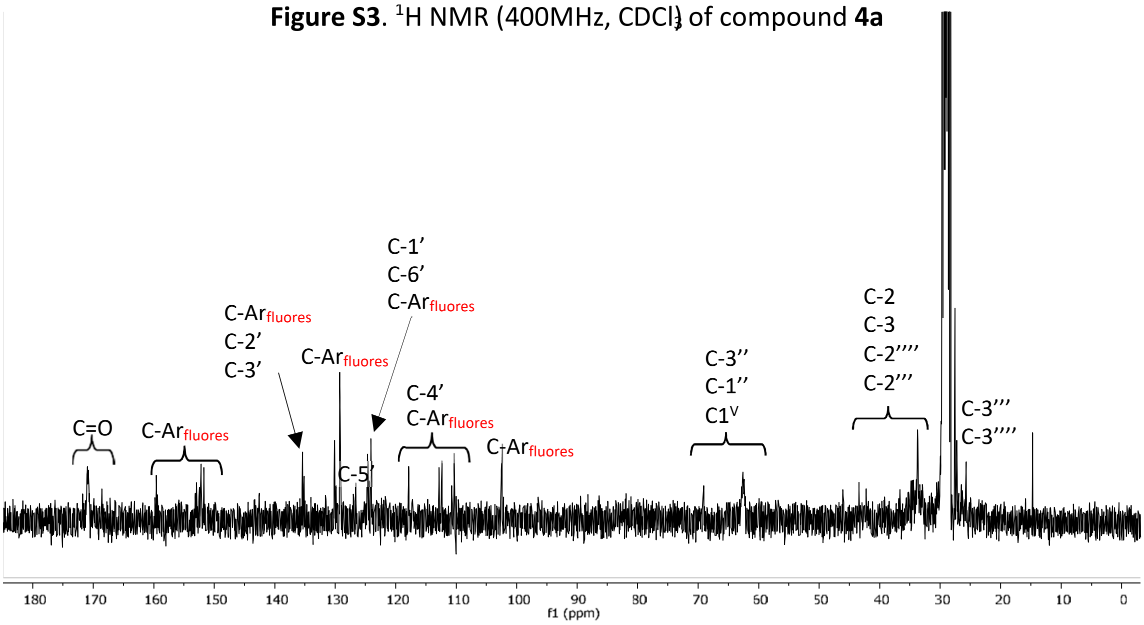

Figure S4. ^{13}C NMR (100MHz, $CDCl_3$) of compound **4a**

3-{[3-(octadecylsulfanyl)propanoyl]oxy}-2,2-bis{[(3-sulfanylpropanoyl)oxy]methyl} propyl 3-[(2,3-dihydroxyphenyl)sulfanyl]propanoate, 4b

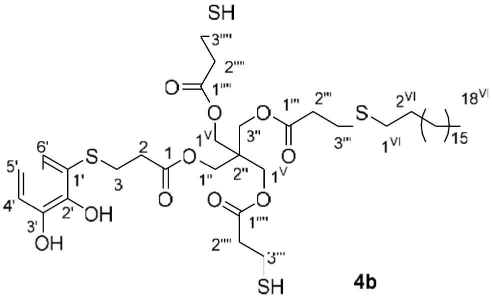

4b

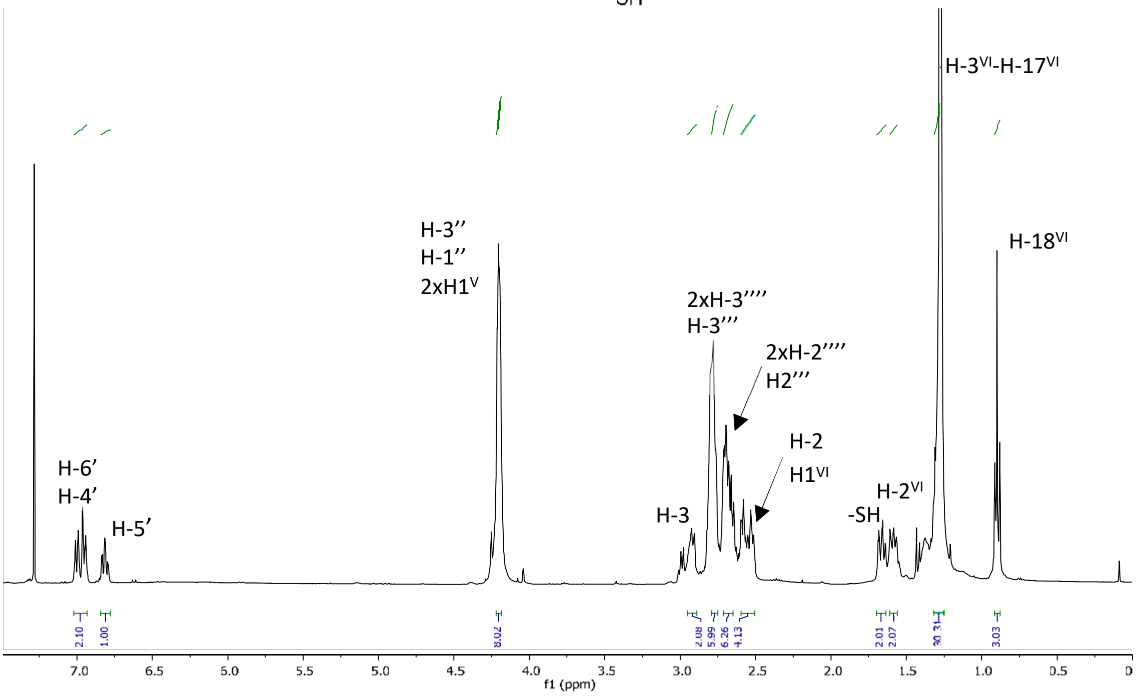

Figure S5. ^{1}H NMR (400MHz, CDCl$_3$) of compound **4b**

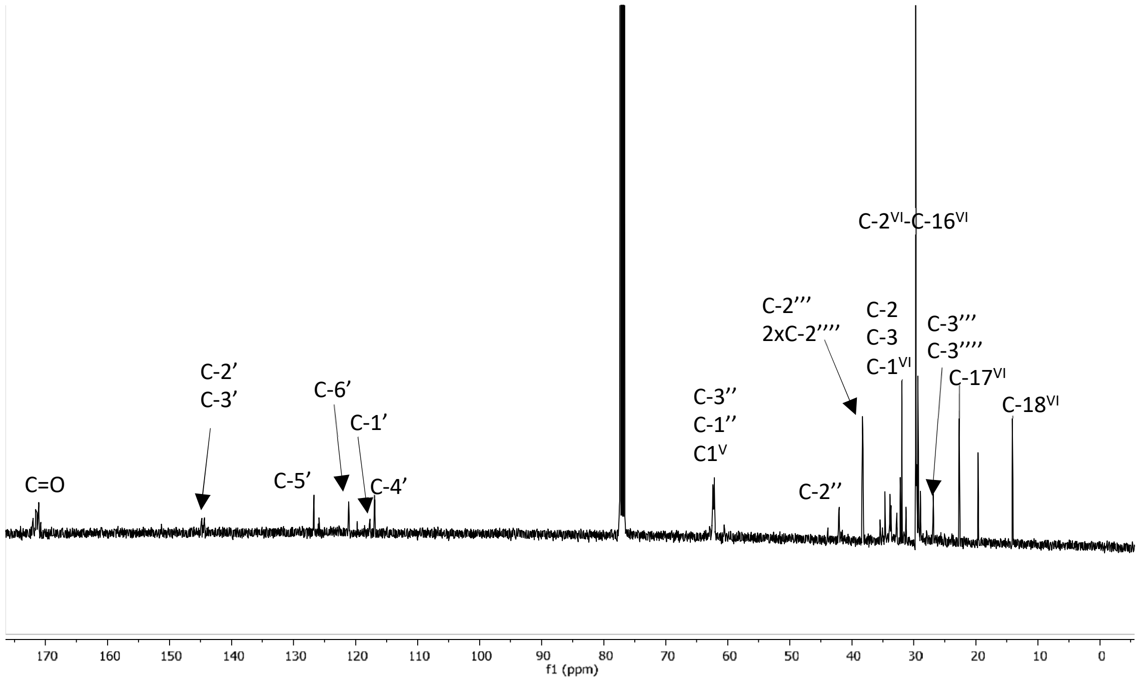

Figure S6. ^{13}C NMR (100MHz, CDCl$_3$) of compound **4b**

3-[(3-sulfanylpropanoyl)oxy]-2,2-bis{[(3-sulfanylpropanoyl)oxy]methyl}propyl 3-(octadecylsulfanyl)propanoate, 5

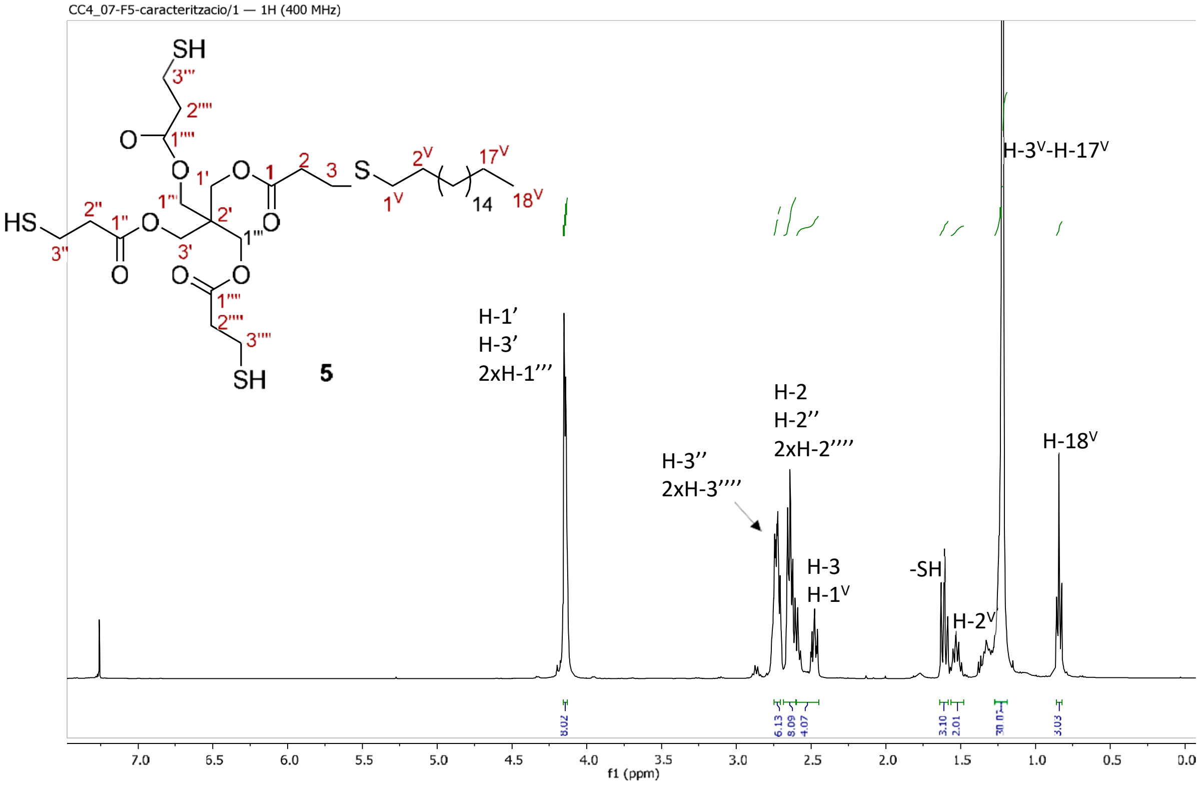

Figure S7. ^{1}H NMR (400MHz, $CDCl_3$) of compound **5**

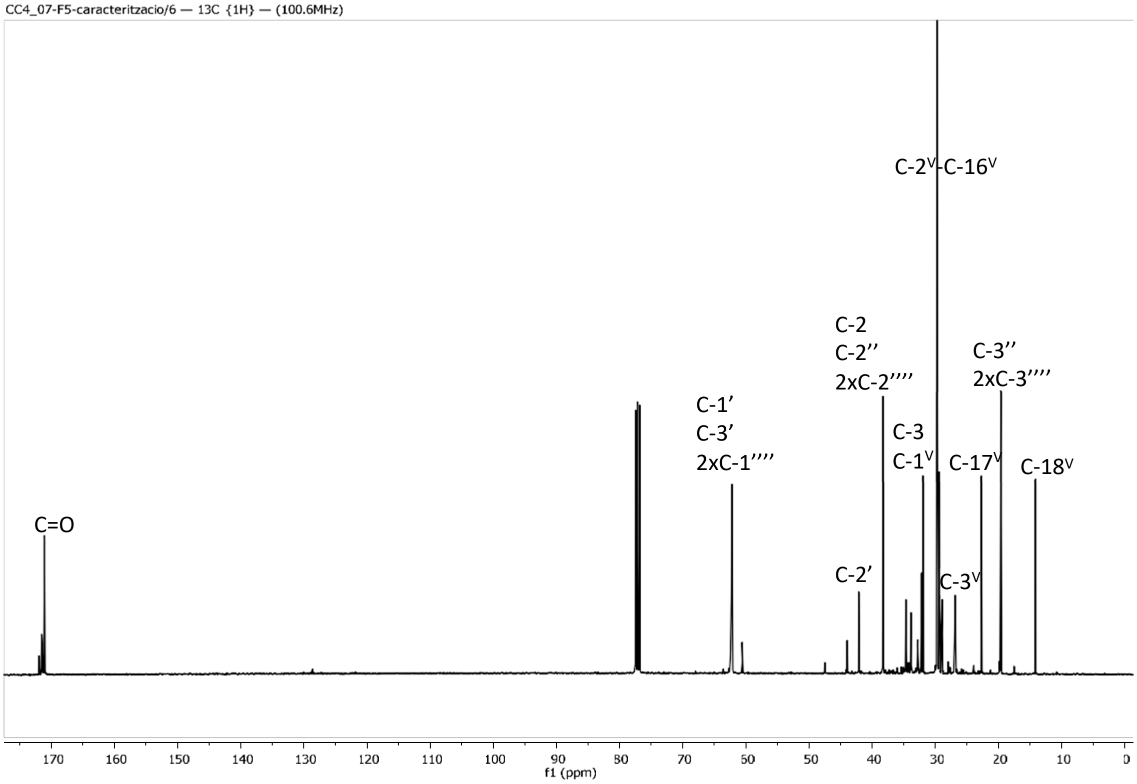

Figure S8. ^{13}C NMR (100MHz, $CDCl_3$) of compound **5**

3-({3-[(3-{[6-hydroxy-9*H*-spiro(3-oxo-isobenzofuran-1-yl)xanthen-3-yl]oxy}-3-oxopropyl)sulfanyl]propanoyl}oxy)-2,2-bis{[(3-sulfanylpropanoyl)oxy]methyl}propyl 3-[(2-phenylethenyl)sulfanyl]propanoate 6a

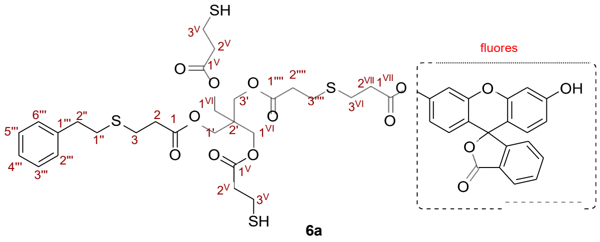

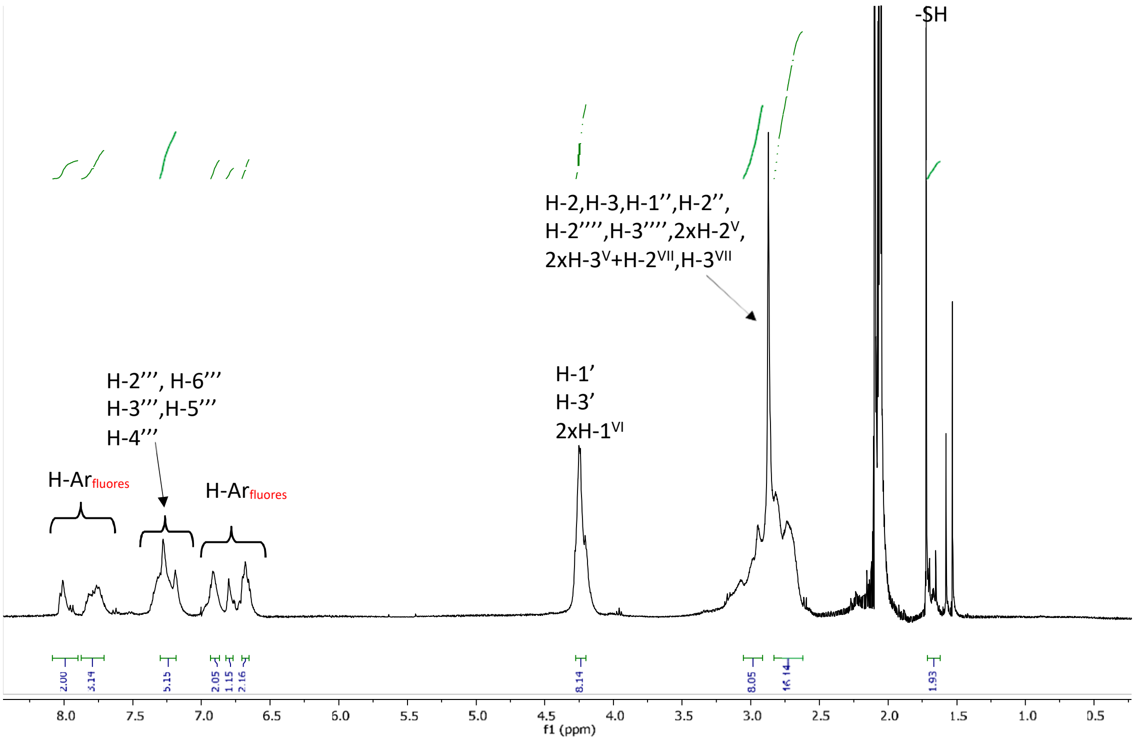

Figure S9. ^{1}H NMR (400MHz, $CDCl_3$) of compound **6a**

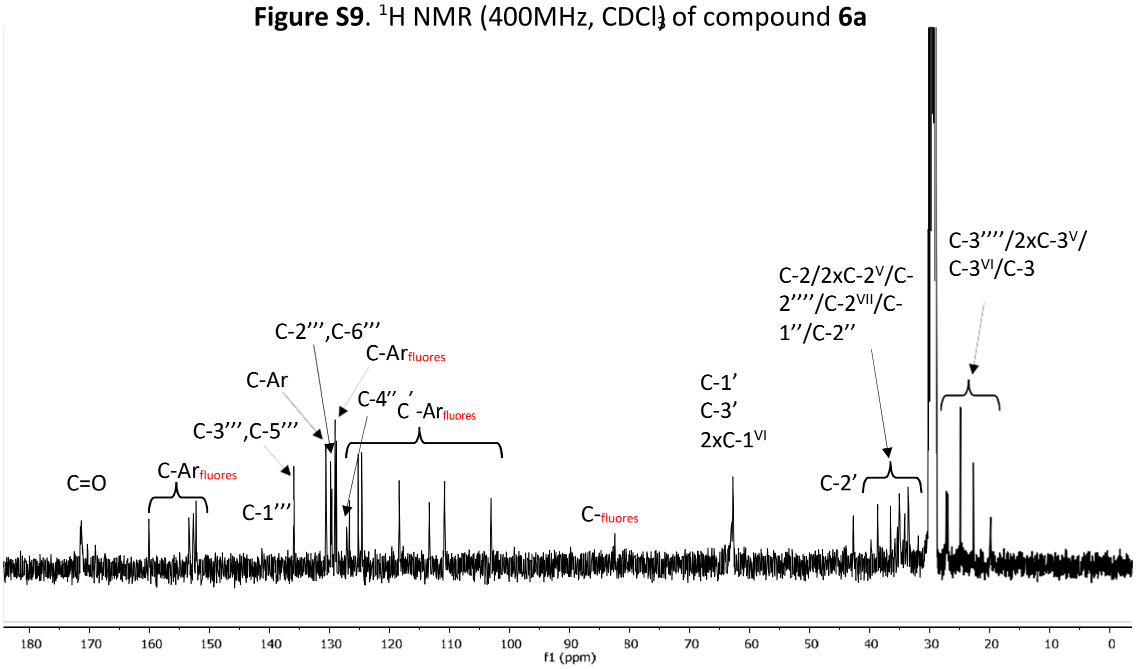

Figure S10. ^{13}C NMR (100MHz, $CDCl_3$) of compound **6a**

3-{[3-(octadecylsulfanyl)propanoyl]oxy}-2,2-bis{[(3-sulfanylpropanoyl)oxy]methyl} propyl 3-[(2-phenylethenyl)sulfanyl]propanoate, 6b

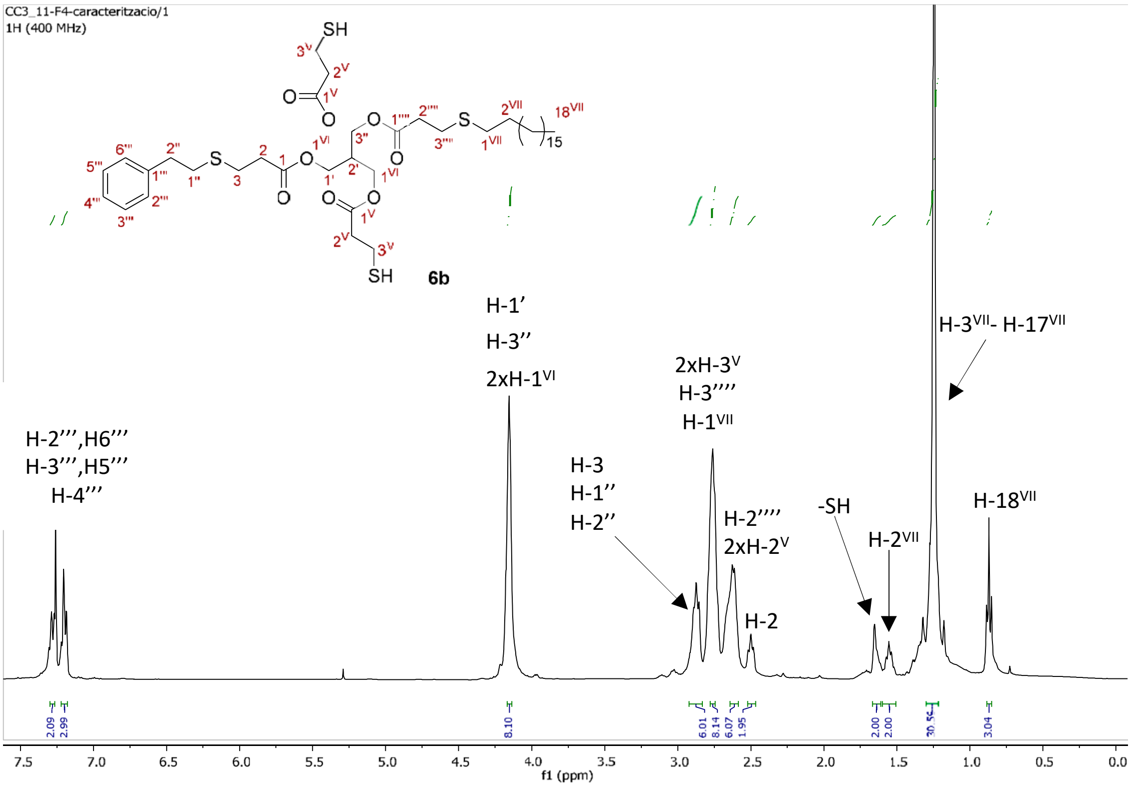

Figure S10. ^{1}H NMR (400MHz, CDCl$_3$) of compound **6b**

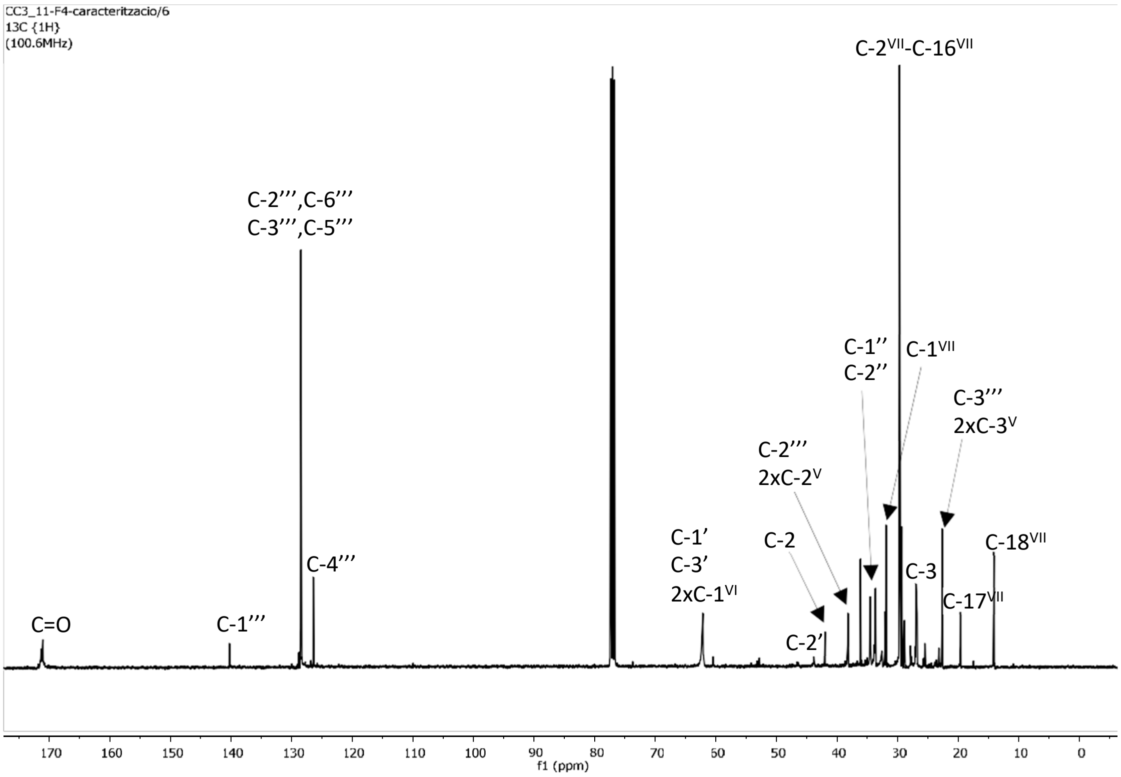

Figure S11. ^{13}C NMR (100MHz, CDCl$_3$) of compound **6b**

3-[(3-sulfanylpropanoyl)oxy]-2,2-bis{[(3-sulfanylpropanoyl)oxy]methyl}propyl 3-[(2-phenylethenyl)sulfanyl]propanoate, 7

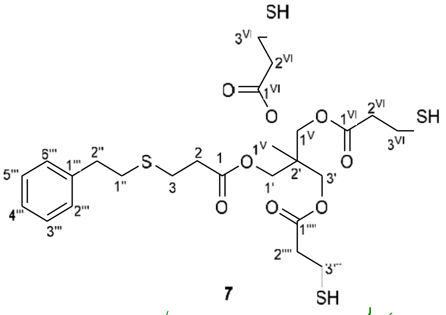

7

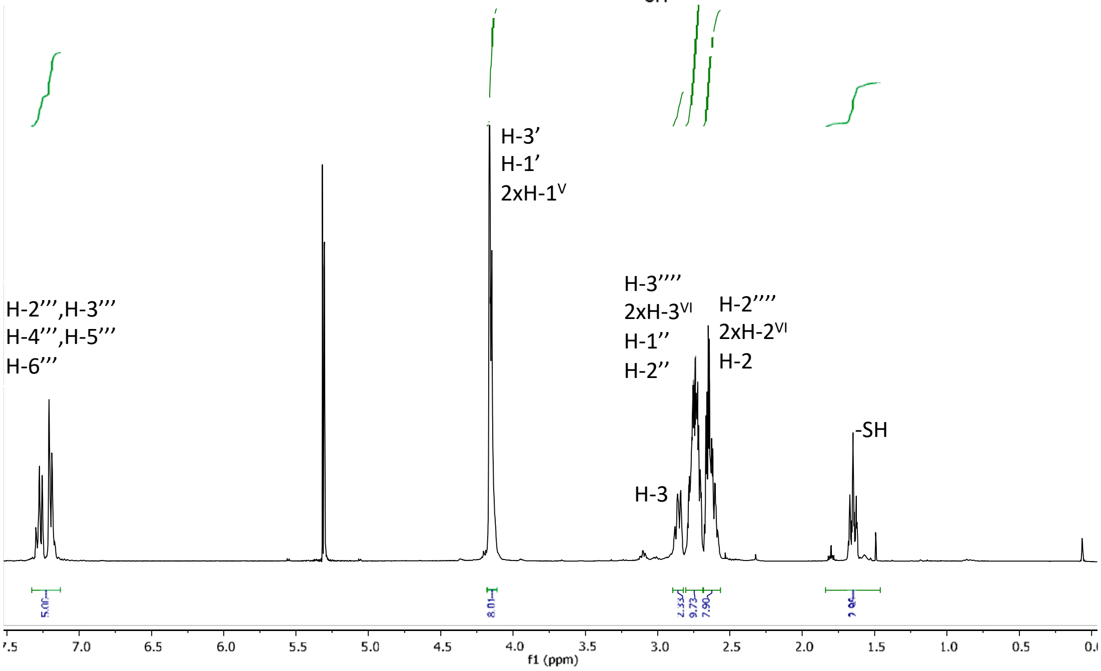

Figure S11. ^{1}H NMR (400MHz, $CDCl_3$) of compound **7**

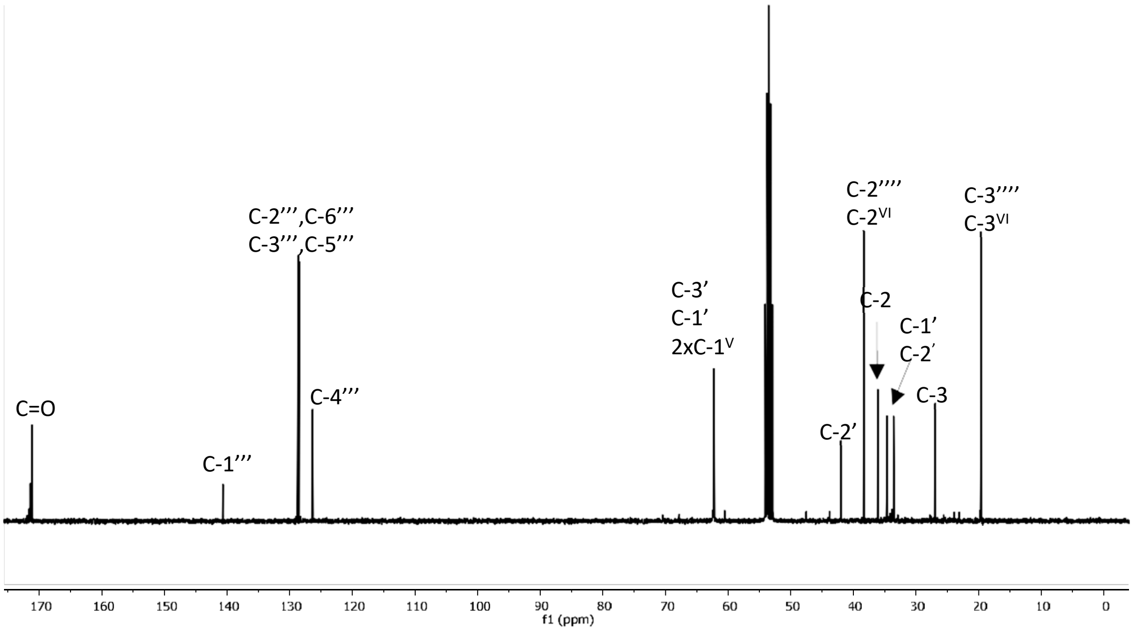

Figure S12. ^{13}C NMR (100MHz, $CDCl_3$) of compound **7**

S5. IR spectra of polymerization reaction products

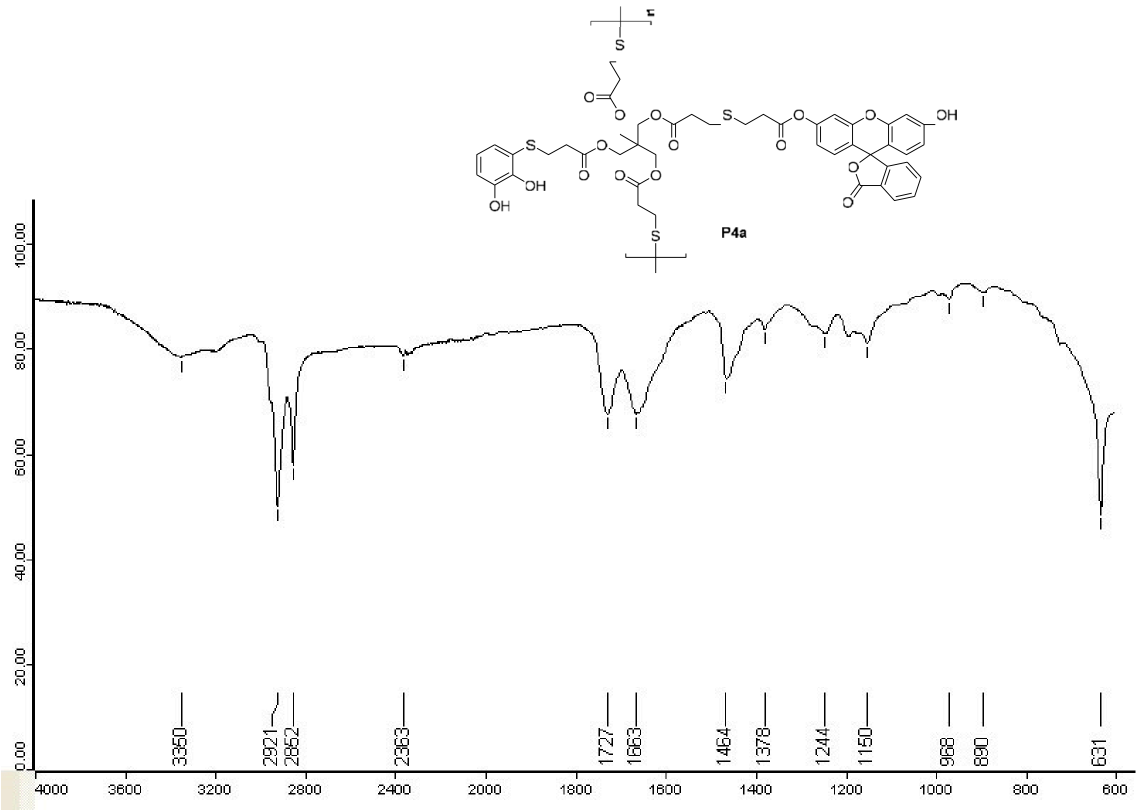

Figure S13. FT-IR spectrum of **P4a**

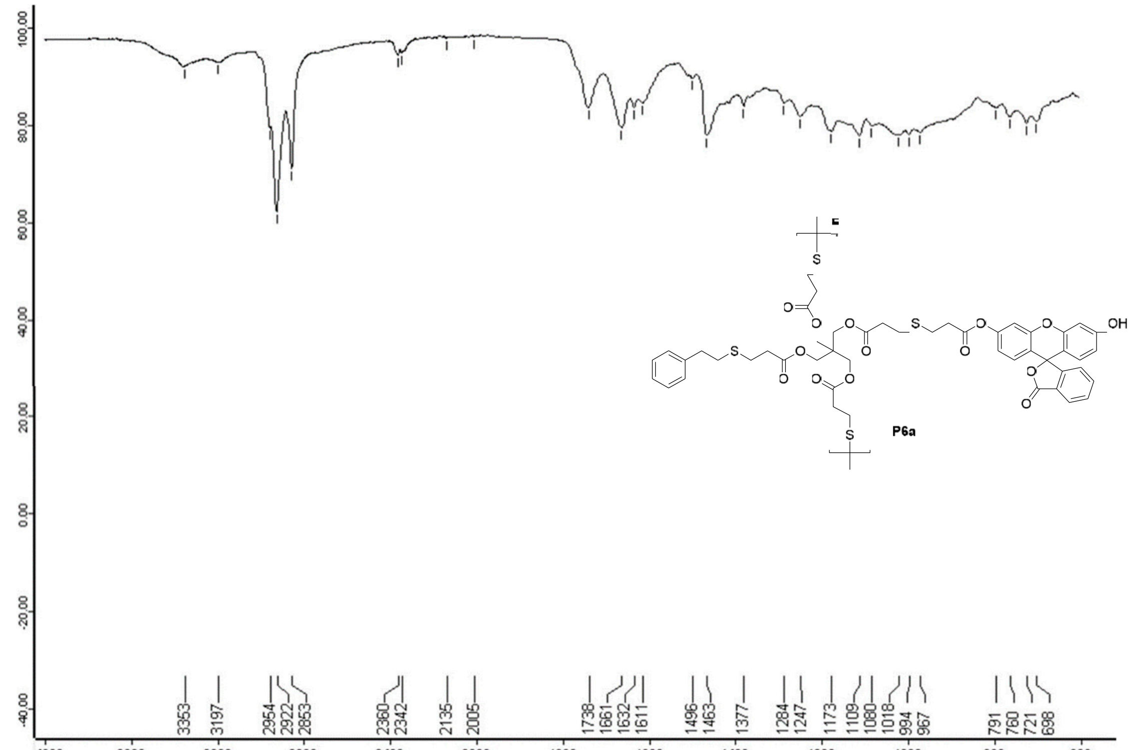

Figure S14. FT-IR spectrum of **P6a**

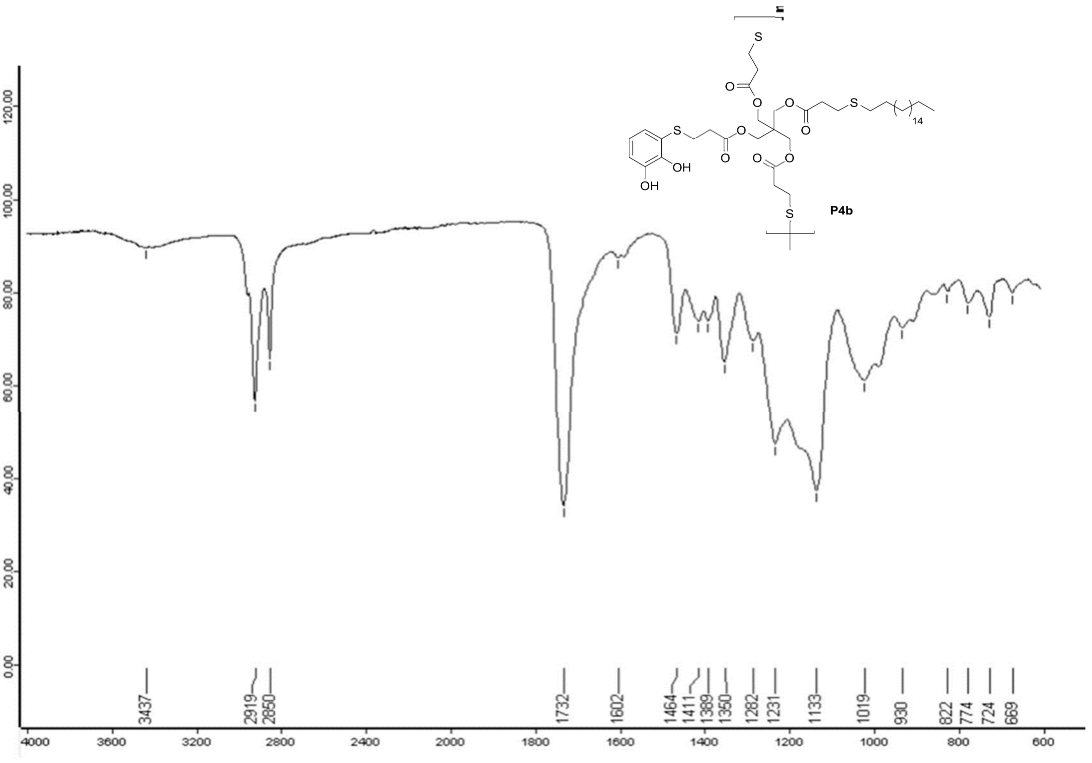

Figure S14. FT-IR spectrum of **P4b**

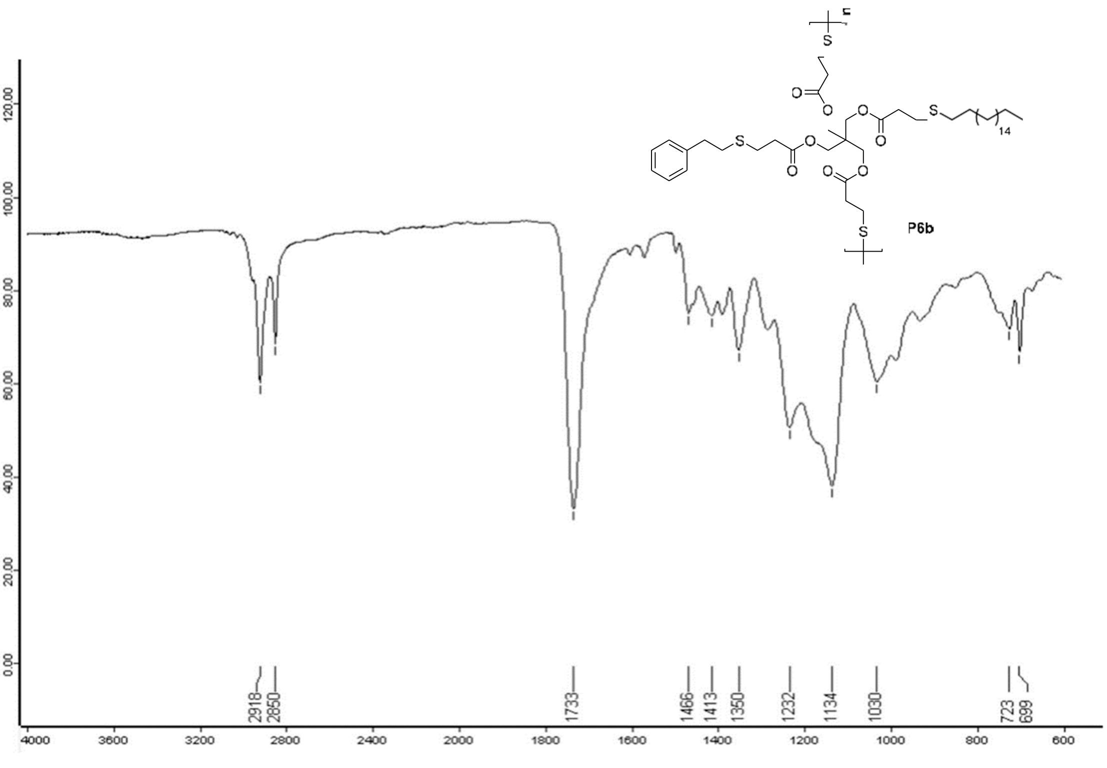

Figure S15. FT-IR spectrum of **P6b**

S6. GPC SPECTRA

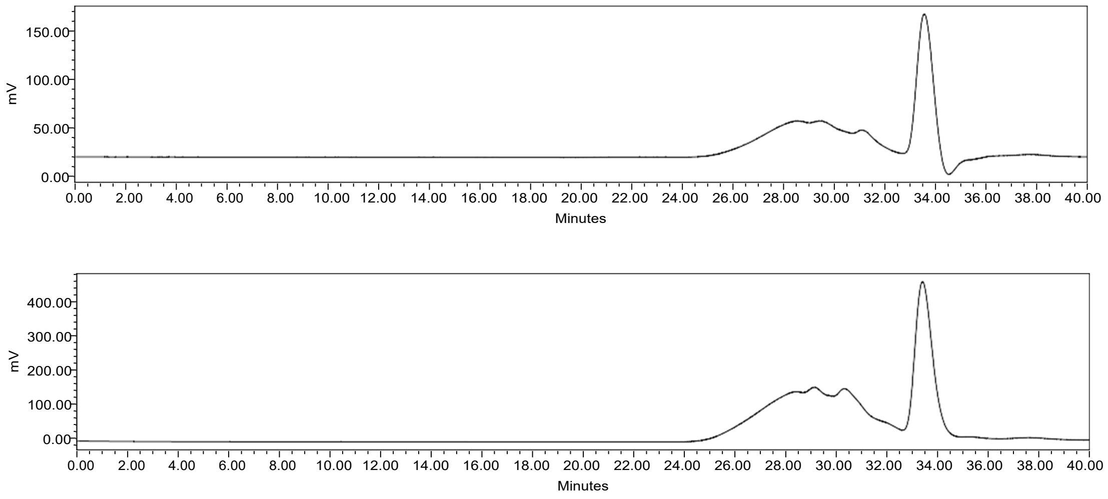

Figure S16. GPC spectra with both IR (top) and UV (bottom) detectors of building block **4b**

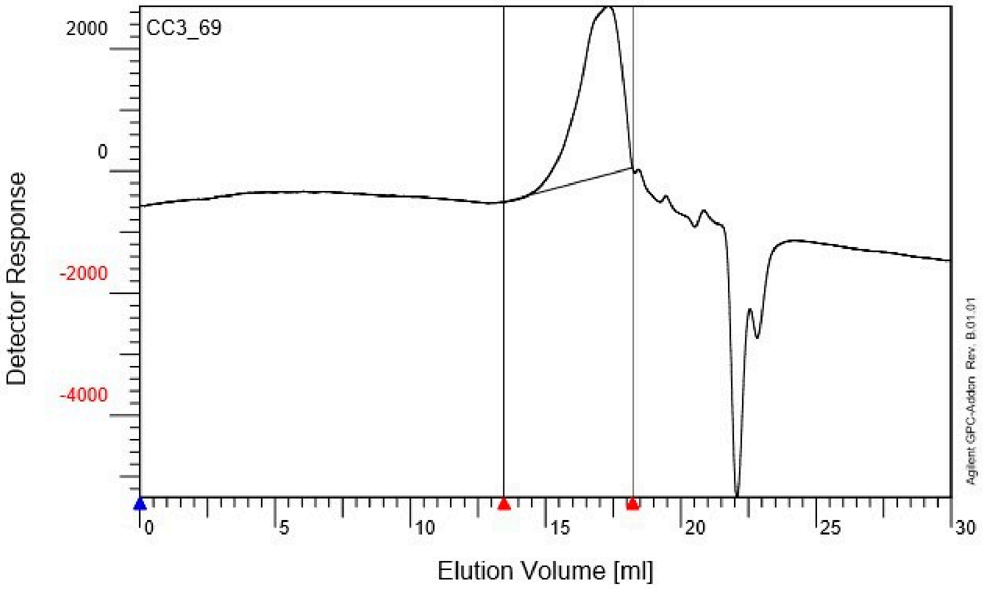

Figure S17. GPC spectrum with IR detector of **P4b**

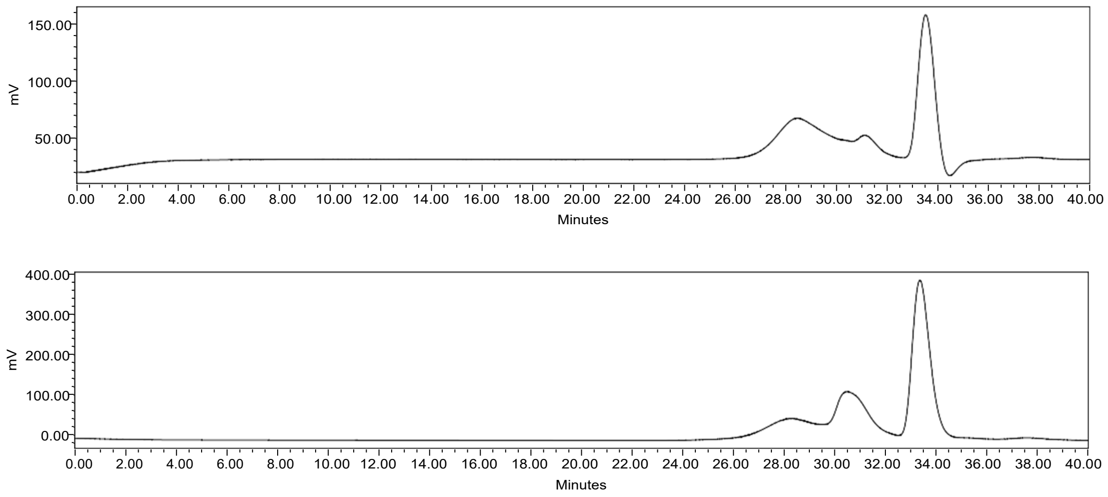

Figure S18. GPC spectra with both IR (top) and UV (bottom) detectors of building block **6b**

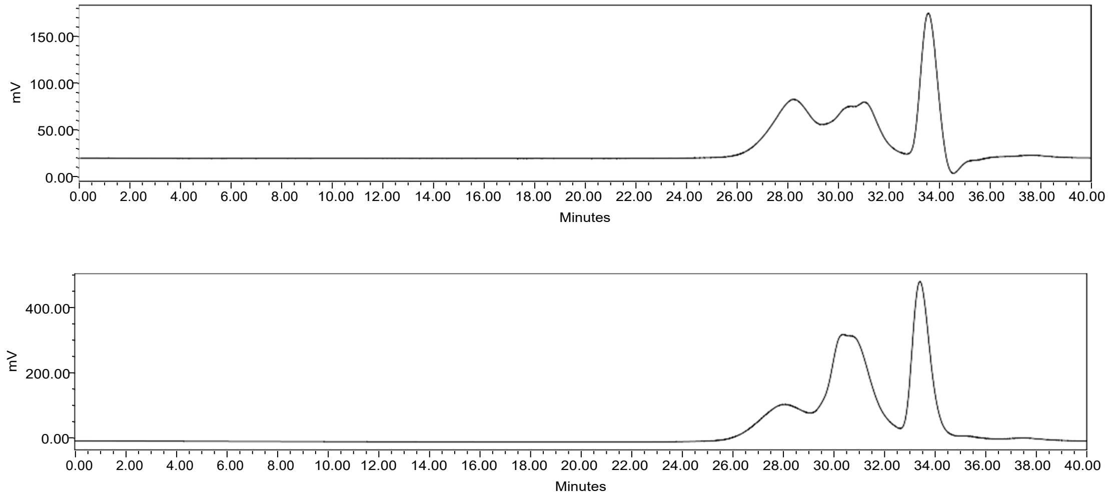

Figure S19. GPC spectra with both IR (top) and UV (bottom) detectors of building block **P6b**

S7. Coating procedure

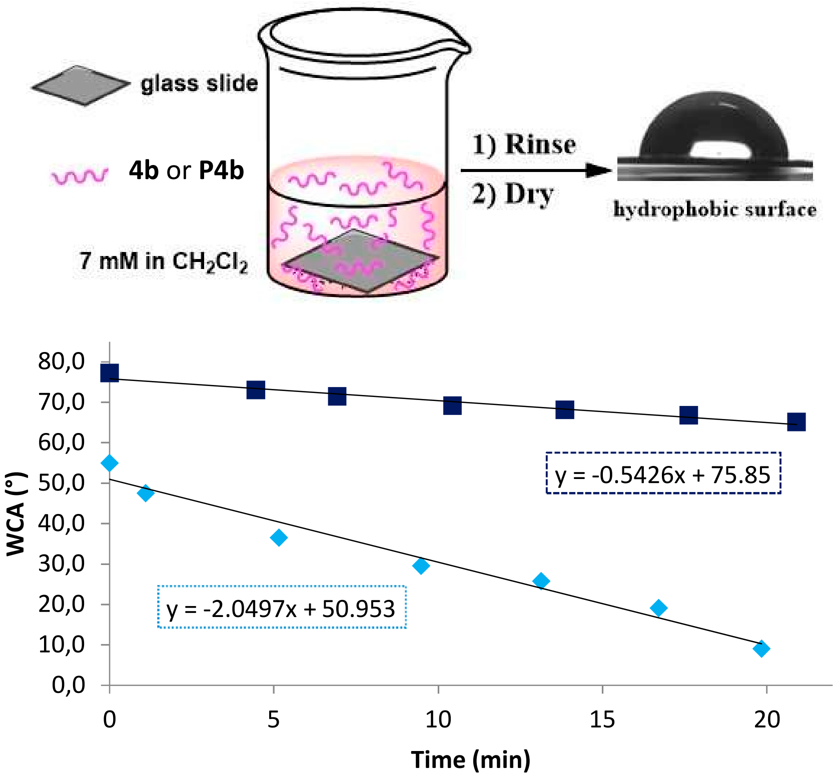

Figure S20. Schematic representation of coating procedure. The glass slide is submerged into the solution for at least 4 h, washed and dried before measuring WCAs. WCA values *vs.* time of **4b**-coated glass slide (light blue) and **P4b**-coated glass slide (dark blue).

S8. EDX SPECTRA

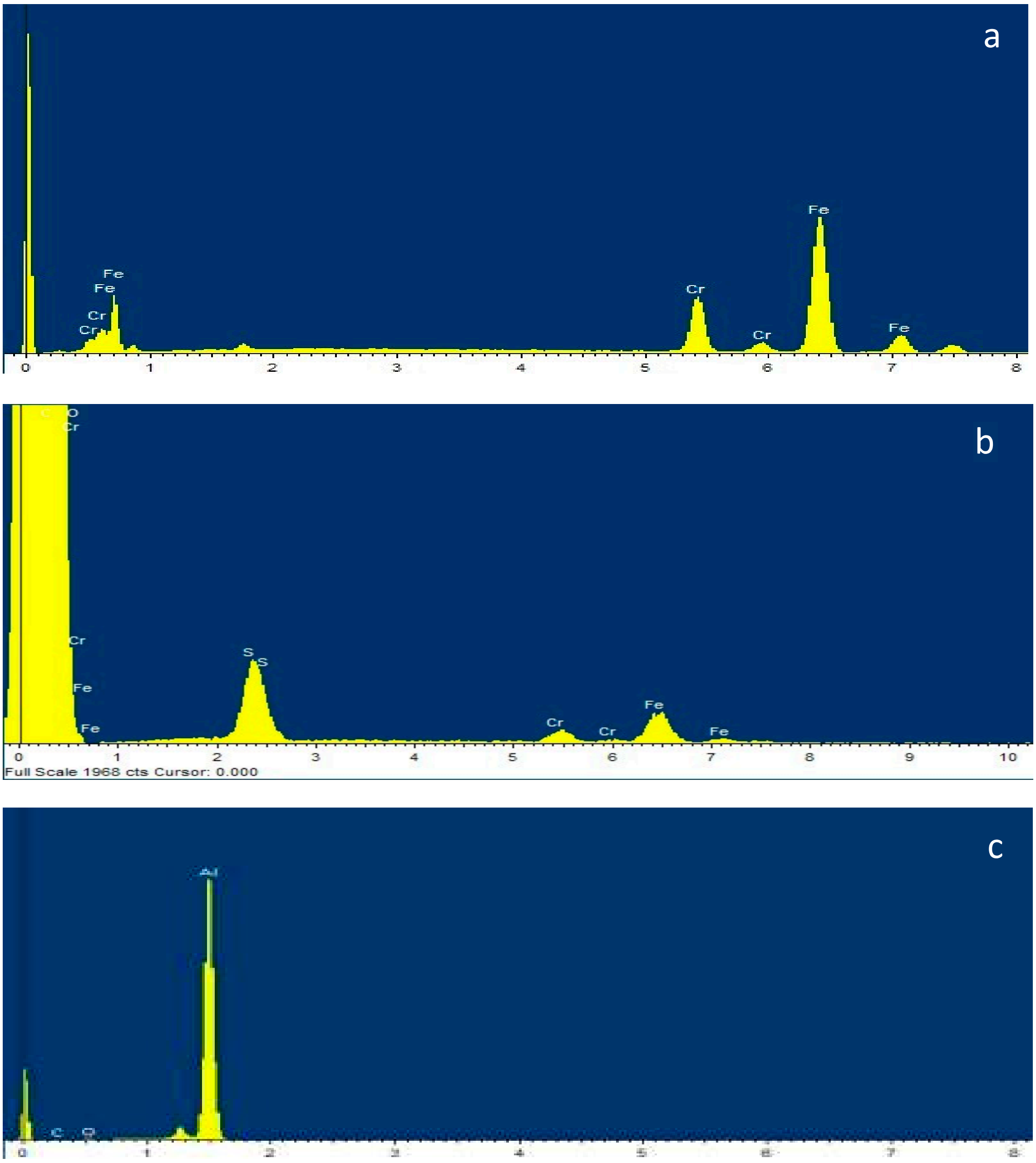

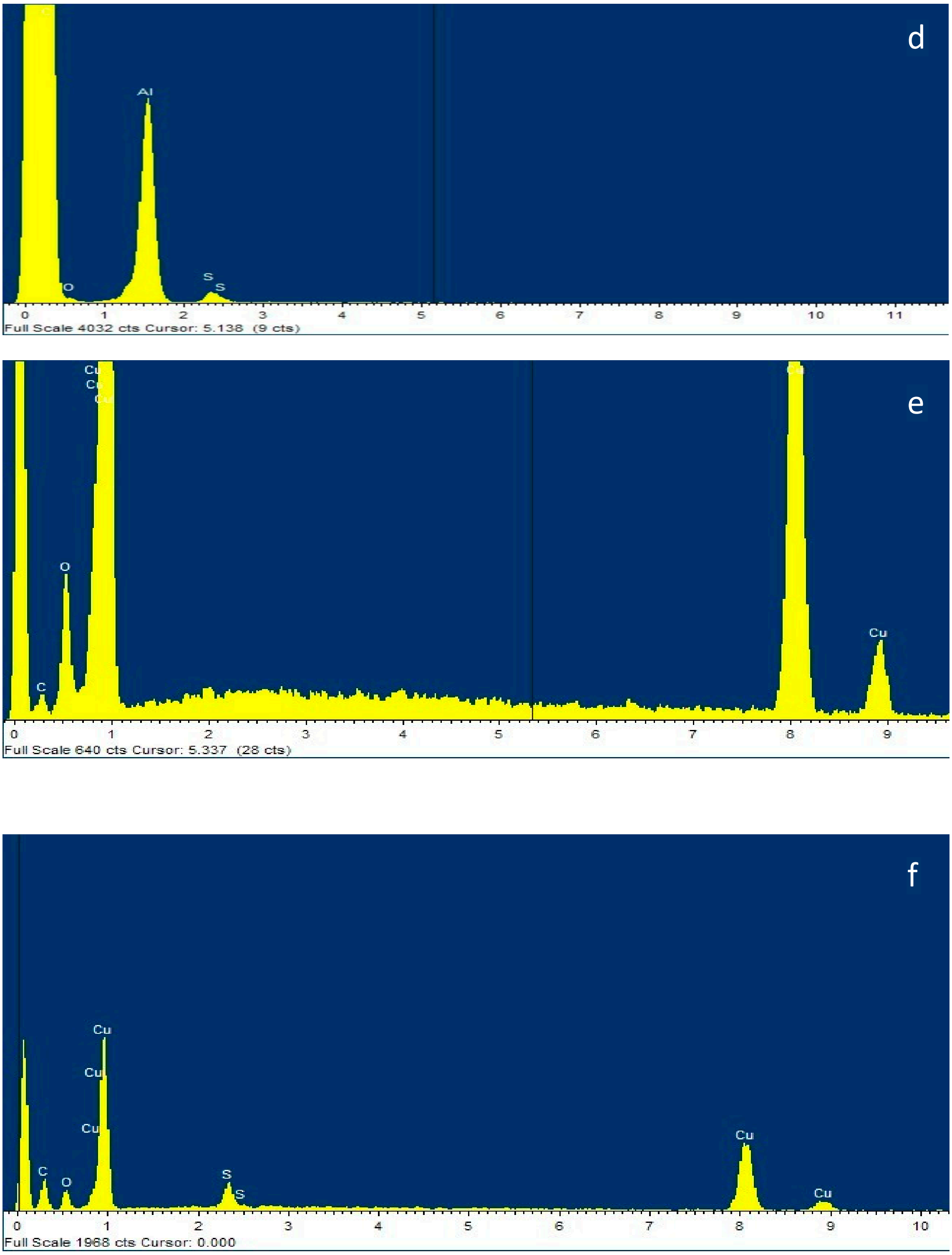

Figure S21. Images from EDX experiments of pristine and coated surfaces with P4b *via ex situ* polymerisation in solution: a) pristine stainless steel (SS), b) SS@**P4b**, c) pristine aluminium, d) Al@**P4b**, e) pristine copper and f) Cu@**P4b**.

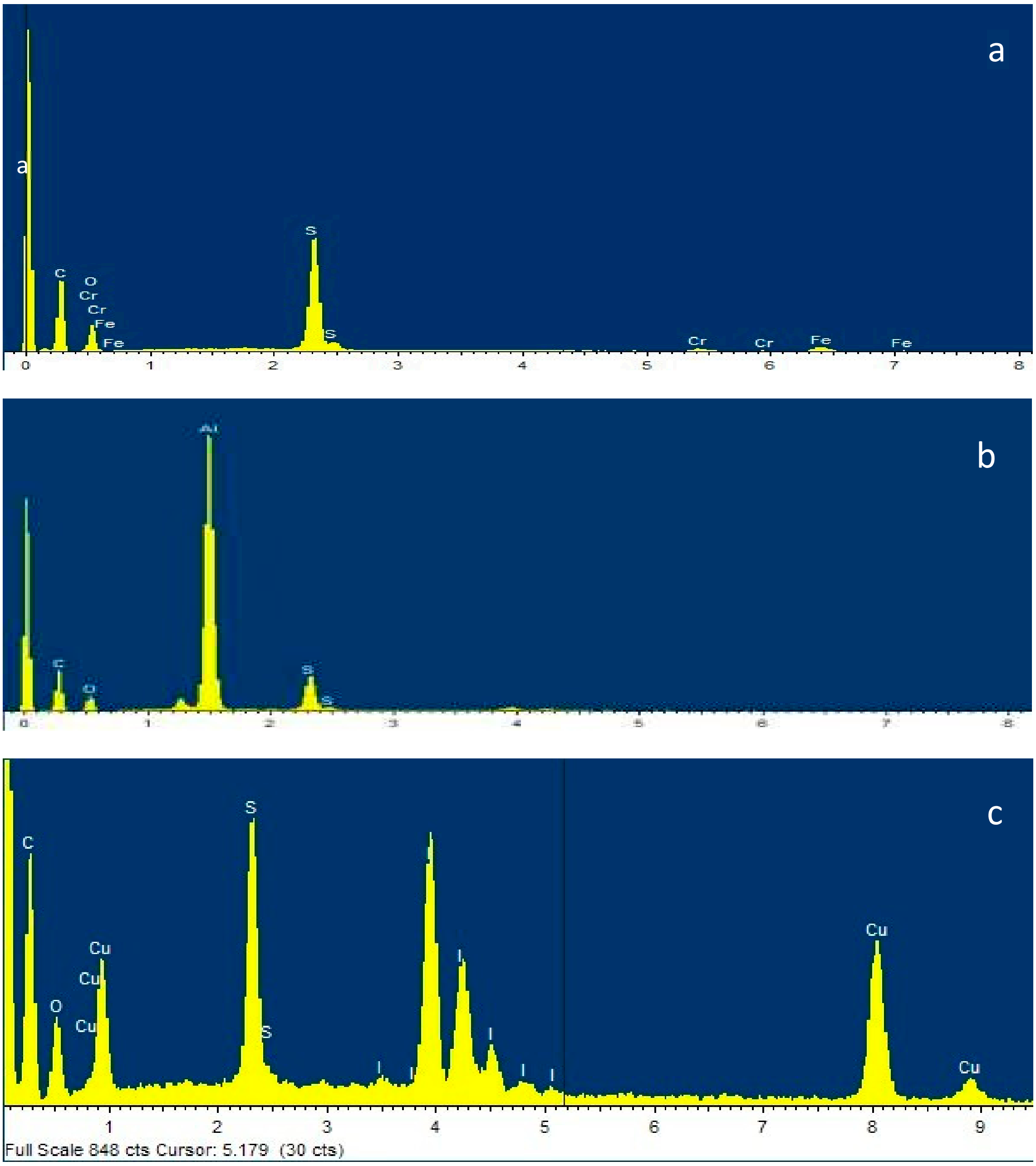

Figure S22. Images from EDX experiments of coated surfaces with P4b *via in situ* polymerisation: (a)SS@**P4b**, b) Al@**P4b** and c) Cu@**P4b**.)

S9. Characterization of coated textiles

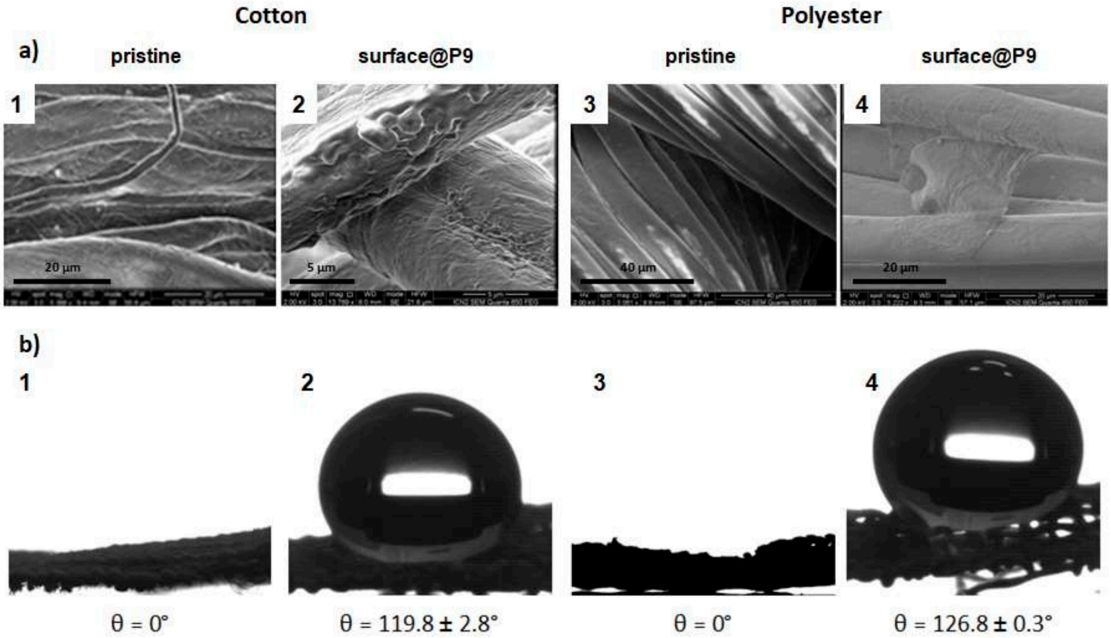

Figure S.23. a) SEM images of pristine fibres (1 and 3), and **P4b**-coated textiles (2 and 4). b) Images from water droplets onto pristine fibres (1 and 3) and **P4b**-coated textiles (2 and 4).
